# The Cytoplasmic MicroRNA‐Production Complex Regulates MicroRNA Sorting to Extracellular Vesicles During Synaptogenesis

**DOI:** 10.1002/advs.78067

**Published:** 2026-09-30

**Authors:** Yuzhou Zeng, Selina Luhmann, Aditi Saha, Marta Orlando, Haider Sami, Timo Glatter, Anna Antoniou

**Affiliations:** ^1^ Department of Old Age Psychiatry and Cognitive Disorders University of Bonn Medical Center Bonn Germany; ^2^ German Center for Neurodegenerative Diseases (DZNE) Bonn Germany; ^3^ Neuroscience Research Center Charité Medical University Berlin Germany; ^4^ Department of Pharmaceutical Sciences Faculty of Life Sciences University of Vienna Vienna Austria; ^5^ Max Planck Institute for Terrestrial Microbiology Marburg Germany

**Keywords:** extracellular vesicle, microRNA sorting, organelle contact sites, RNA granule, RNA‐binding protein, synapse formation

## Abstract

Extracellular vesicles (EVs) have emerged as novel players of cell communication, in part via the transfer of microRNA (miRNA) cargo to recipient cells. Although several RNA‐binding proteins (RBPs) were shown to shuttle miRNAs to EVs, how they are loaded with miRNA is unknown. This study demonstrates that the core accessory protein of the cytoplasmic miRNA production machinery TRBP interacts with several EV‐RBPs and differentially regulates miRNA secretion in small EVs. TRBP‐regulated EV‐miRNAs were highly enriched in a Syncrip‐recognition sequence motif. Moreover, we show that Syncrip associates with TRBP via its C‐terminal domain at the surface of the endoplasmic reticulum (ER), a nucleation site of the miRNA production machinery. The recruitment of Syncrip to ER‐associated TRBP was regulated by ER‐endosome membrane contact sites (MCS), whereby increased MCS formation promotes Syncrip loading to endosomes as well as Argonaute 2‐positive RNA granules. Functionally, this pathway is important for the regulation of synapse formation in developing neurons, as expression of miRNA‐ or TRBP‐binding deficient mutants of Syncrip differentially regulated synapse formation in EV‐donor and recipient neurons. Overall, this work highlights an important role of TRBP as a gatekeeper for microRNA sorting, which could be crucial for the fine‐tuning of neuronal circuits during postnatal development.

## Graphical abstract

The mechanisms of microRNA cargo sorting to extracellular vesicles, important mediators of cell communication, are not fully understood. This study reveals that the microRNA‐production machinery acts as a ‘hub’ for the sorting of microRNAs to extracellular vesicles, a process regulated by dynamic organelle interactions. In neuronal cells, this pathway is crucial for balancing synaptic strength between secreting and recipient neurons.

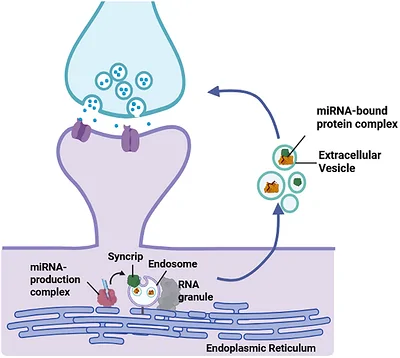

## 1 Main

Extracellular vesicles (EV) are membraneous vesicles secreted by most cells that contain lipid, protein, and RNA cargo. Many publications have shown that small EVs, such as those originating from multi‐vesicular endosomes (also known as exosomes), contain microRNA (miRNA) cargo that can, in some cases, be delivered to recipient cells, thereby regulating a diverse set of biological processes [1, 2]. In the nervous system, miRNAs are important regulators of local translation at sites distant from the cell soma, and are thus crucial for neuronal circuit formation and synaptic plasticity [3, 4]. The inter‐cellular delivery of functional miRNAs may therefore be especially relevant in neurons and other highly differentiated cells, which rely on local translational control in discrete compartments [5]. We indeed previously showed that neuronal small EVs contribute to synapse formation downstream of BDNF (*brain‐derived neurotrophic factor*)‐TrkB signaling, at least in part via the specific sorting of miRNA cargo and delivery to recipient neurites [6]. Nevertheless, the mechanisms for miRNA selection and sorting to EVs are still elusive, especially in neurons.

A number of RNA‐binding proteins (RBPs) were shown to regulate the secretion of miRNAs in EVs in non‐neuronal cells. For example, hnRNPA2B1 was one of the first RBPs shown to mediate EV‐miRNA secretion in its sumoylated form, particularly for miRNA containing a ‘GGAG’ motif at their 3’ end [7]. This motif was termed the ‘EXO’ motif, and was shown to be enriched in EV‐associated, compared to cell‐retained (‘CELL’) miRNAs, in microarray analysis [7]. Since then, several other RBPs such as Ybx‐1 [8, 9] and Syncrip (also known as hnRNPQ) [10, 11], were shown to bind to miRNAs via different motifs and escort them to EVs. Furthermore, a study that profiled intracellular and EV‐associated miRNAs from cells of different origins revealed that cells employ multiple ‘EXO’ and ‘CELL’ motifs, in a cell type‐dependent manner [12]. In addition to RBP interactions, intracellular organelles were shown to play a role in the regulation of RNA sorting to EVs [8, 13]. For example, the formation of membrane contact sites (MCS) between the endoplasmic reticulum (ER) and other organelles such as endosomes by the linker protein VAP‐A was shown to be important for the secretion of RNA‐enriched EVs [13]. In addition to organelle contacts, the formation of liquid‐liquid phase condensates containing RNA is thought to be important for RNA sorting into EVs [8]. Whether and how these sorting mechanisms are coupled and how EV‐RBPs are loaded with miRNA are, however, still unclear.

MiRNA production is a multi‐step process that is regulated by core and accessory RBPs [14]. Following their export from the nucleus, precursor miRNAs (pre‐miRNA) are cleaved by the RNase III Dicer into an ∼ 22 nucleotide (nt) miRNA duplex that can be loaded onto Argonaute proteins (Ago), the major catalysts of miRNA‐mediated translational repression by the RNA‐induced silencing complex (RISC). The Dicer interacting protein TRBP (*transactivation response element RNA‐binding protein*) was shown to enhance cleavage fidelity and accuracy by promoting a cleavage‐competent ‘open’ Dicer conformation [15, 16, 17, 18]. In addition, it has been suggested that TRBP aids the loading of the cleaved miRNA duplex to Ago through the formation of a ternary complex consisting of Dicer, TRBP, and Ago, called the RISC loading complex (RLC) [19]. The TRBP‐containing RLC complex was shown to preferentially nucleate on the cytoplasmic surface of the ER [20, 21]. We herein report that TRBP differentially regulates miRNA secretion in neuronal small EVs, likely via interactions with RBPs previously implicated in EV‐miRNA secretion. TRBP‐regulated miRNAs were highly enriched for a 6 nucleotide‐long oligomer (6‐mer) motif containing a previously reported Syncrip recognition sequence. Interestingly, MCS between the ER and endosomes dynamically regulated the recruitment of Syncrip to ER‐associated TRBP‐RLC, as well as endosomes and Ago2‐positive RNA granules, indicating a mechanism for the dynamic regulation of the sorting of *de novo* produced miRNAs to EV‐secreted RBPs. Finally, we demonstrate that Syncrip interactions with TRBP or miRNAs is functionally important for synapse formation in EV donor and recipient neurons, a process that relies on the local regulation of translation. Overall, this work provides insight into the mechanisms underlying the loading of miRNA to EV‐secreted RBPs by a major component of cytoplasmic miRNA production, which may have broad functional implications and could ultimately aid the development of EV‐ and RNA‐based therapeutic approaches.

## 2 Results

### 2.1 The Dicer Co‐Factor TRBP Interacts With RBPs Secreted in EVs

Several RBPs were shown to be essential for the activity and sorting of RNAs to EVs [2]. TRBP is a major component of the RLC, which cleaves pre‐miRNAs and loads mature miRNA onto Ago and the RISC. To investigate whether TRBP is implicated in miRNA sorting by RBPs, we performed mass spectrometry of primary neuronal cell lysates subjected to immunoprecipitation (IP) with an anti‐TRBP antibody or IgG control. Here, we observed a substantial fraction of overlapping proteins between TRBP IP and neuronal EV fractions, based on our previously published proteomics dataset of primary neuronal EVs [22] (Figure 1A). Protein network analysis of overlapping proteins revealed the enrichment of mRNA and miRNA binding proteins, as well as constituents of the cytoskeleton (Figure 1A and Figure S1A and Table S1). We then validated the interaction between TRBP and RBP candidates, including YBX‐1, hnRNPA2B1, and Syncrip, which were previously shown to play a role in miRNA sorting in EVs [7, 9, 10], in N2a neuroblastoma cells (Figure 1B–E and Figure S1B,C). IP experiments in N2a cells expressing GFP, GFP‐TRBP WT, or the Dicer binding‐deficient deletion mutant of TRBP (GFP‐TRBP‐∆C) showed that both TRBP‐WT and ‐∆C interact with Syncrip and the two known isoforms of YBX‐1, suggesting that they could potentially associate in complex with the RLC (Figure 1B,C). In addition, we observed the interaction between myc‐tagged hnRNPA2B1 and GFP‐TRBP and its orthologue PACT in N2a cells (Figure S1B). However, hnRNPA2B1 could no longer co‐sediment with the bead‐antibody fraction containing GFP‐TRBP IPs upon RNase A treatment, suggesting that the hnRNPA2B1‐TRBP interaction is RNA‐dependent (Figure 1D and Figure S1C). This is in contrast to the Syncrip‐TRBP interaction, where GFP‐Syncrip seems to bind more strongly to myc‐tagged TRBP in the absence of RNA, as the majority of Syncrip was detected in the bead‐antibody fraction following RNase A treatment (Figure 1D,E). Following validation of TRBP‐RBP interactions in N2a cells, we further verified the endogenous interaction between TRBP and Syncrip using IP in primary cortical neurons and observed that they partially co‐localize in proximity to Synaptophysin‐positive synapses in dendrites of mature hippocampal neurons (Figure 1F,G).

**FIGURE 1:**
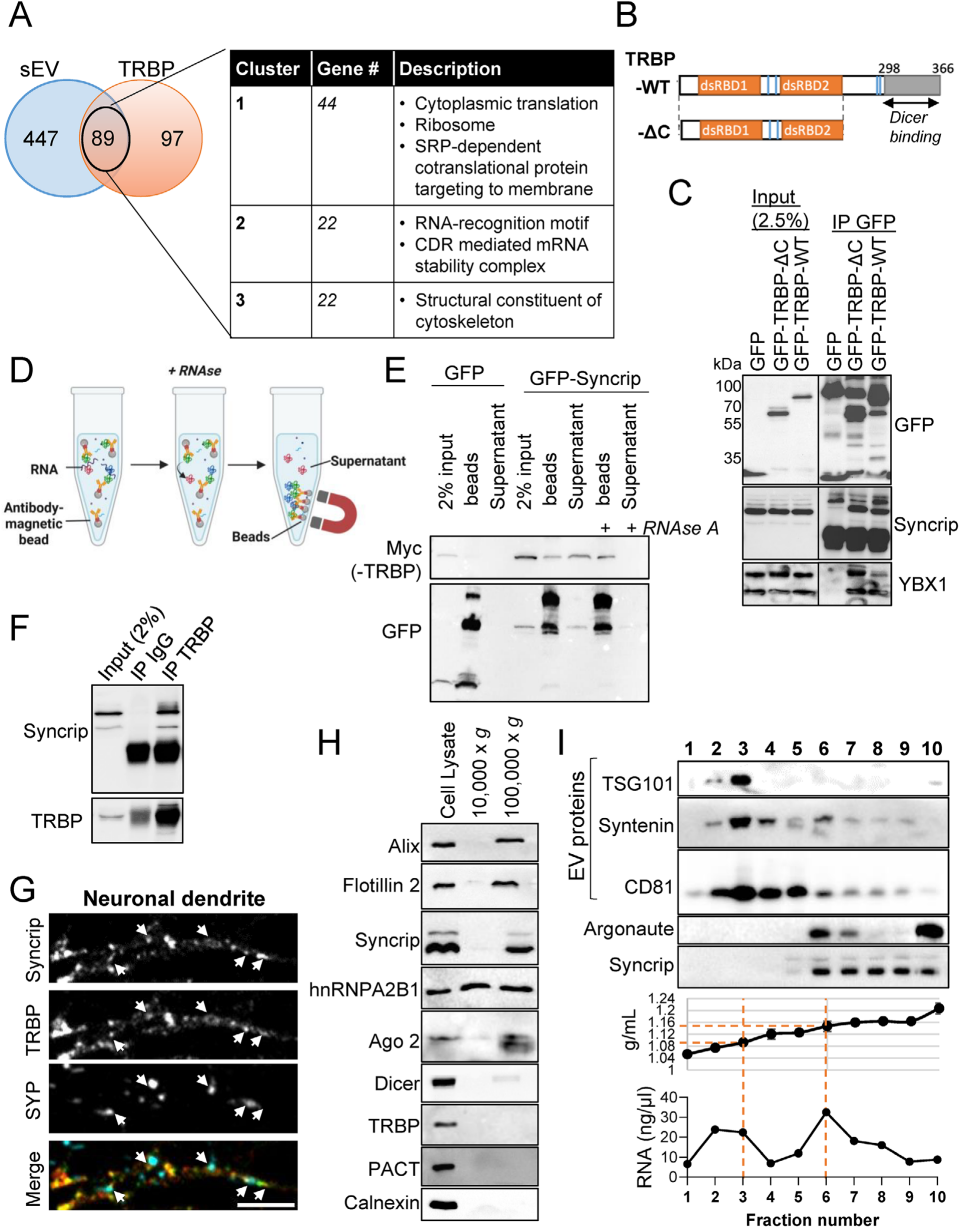
The Dicer co‐factor TRBP interacts with RBPs secreted in EVs. (A) Venn diagram of proteins found in neuronal small EVs and TRBP immunoprecipitates (IP), identified by mass spectrometry. Table below indicates node descriptions following k‐means *STRING* clustering of overlapping proteins. (B) Primary sequence of TRBP WT and ∆C. (C) TRBP interacts with Syncrip and YBX‐1 independently of Dicer binding. N2a cells were transfected with GFP, GFP‐TRBP‐WT or GFP‐TRBP‐∆C and IP was performed using an anti‐GFP antibody. 100 kDa bands in IP lanes likely correspond to GFP antibody used in IP, or non‐specific bands. (D) Depiction of RNase A digestion experiment. RNA IPs were digested with RNAse A prior to magnetic isolation of IP pellets and supernatants. RNA‐dependent interacting protein shown in red, RNA‐independent interacting protein in blue. Created in BioRender (Antoniou, A. (2026) https://BioRender.com/2ku818i). (E) Myc‐TRBP binds to GFP‐Syncrip in an RNA‐independent manner. GFP IP were treated with RNase A (0.1 µg/mL, 10 min, 37°C) or control as shown in D. (F) Endogenous TRBP and Syncrip interact in primary cortical neurons (7DIV). G) Confocal images of 18DIV hippocampal neuron dendrites. Arrows point to overlapping TRBP (red) and Syncrip (green) puncta in proximity to synaptophysin (SYP, cyan), scale bar; 15 µm. (H) Western blot analysis of neuronal EVs. EVs were isolated by ultracentrifugation (UC) from primary cortical neurons (5 DIV). (I) Western blotting of light and dense EV fractions. EV from primary cortical neurons (6 DIV) isolated by 100 000 x*g* UC were loaded underneath an iodixanol density gradient and subjected to centrifugation at 120 000 x*g*, 18 h. Shown at the bottom is the density of each fraction and the RNA concentration.

We then investigated whether the RLC and interacting RBPs are secreted in neuronal small EVs isolated using ultracentrifugation (UC). Although we did not observe the secretion of RLC proteins Dicer, TRBP, or PACT in EVs, we detected Syncrip, as well as Ago2 in the 100 000 x*g* EV pellet derived from primary cortical neurons (Figure 1H). We further observed that in contrast to Syncrip, which was enriched in the 100 000 x*g* UC pellet, hnRNPA2B1 was enriched in both the 100 000 x*g* and 10 000 x*g* pellets, the latter mainly consisting of medium‐sized EVs in neurons [6] (Figure 1H and Figure S1D). Importantly, overexpression of GFP‐TRBP WT or the Dicer‐deficient binding mutant TRBP‐∆C in N2a cells did not affect the secretion of Syncrip or hnRNPA2B1 in small‐ or medium‐sized EVs (Figure S1D), although this does not exclude potential changes in RBP abundance in EV sub‐types or their specific functions. To investigate whether Syncrip and Ago are present in distinct EV sub‐types, we performed further fractionation of 100 000 x*g* EV pellets derived from primary cortical neurons using density gradient centrifugation. Here, we observed the co‐sedimentation of Ago and Syncrip in Syntenin‐positive dense EV fractions (fractions 6–7) that also contained RNA, as well as in the non‐vesicular faction 10 (Figure 1I). This result is in agreement with a previous study in non‐neuronal cells, which reported that small dense EVs contain miRNA‐binding proteins, such as Ago [13]. Taken together, this data suggests that TRBP can interact with EV‐RBPs like Syncrip, hnRNPA2B1, and YBX‐1, likely as part of the RLC, but is not itself secreted in small or medium‐sized EVs.

### 2.2 TRBP Differentially Regulates the Secretion of miRNAs in Neuron‐Derived Small EVs

To investigate the role of TRBP in EV‐miRNA secretion, we performed siRNA‐mediated knockdown (KD) of TRBP in primary cortical neurons (Figure S2A–C) and subjected cell lysates and corresponding small EVs to small RNA sequencing. To ensure better isolation of EVs from non‐vesicular fractions, EVs were isolated using size exclusion chromatography (SEC) and EV‐enriched fractions (as in ref [6]). were pooled together and further concentrated using UC (SEC‐UC). Small RNA sequencing data revealed the differential expression of several miRNAs in neuronal cell lysates (CL) and small EVs upon TRBP KD, with particularly pronounced changes in EV fractions (Figure 2A–C and Figure S2D). Furthermore, there was little correlation in TRBP‐dependent changes in miRNA abundance between EVs and CL (Figure 2D), suggesting selective mechanisms for miRNA sorting into small EVs. To investigate putative functions of significantly regulated miRNAs, we performed gene ontology analysis of predicted targets using neuronal transcriptome data as background. For both EV and CL miRNAs, predicted targets were associated with biological processes related to synapse and dendrite development, synapse organization, and synaptic activity (Figure 2E and Figure S2E and Tables S2 and S3).

**FIGURE 2:**
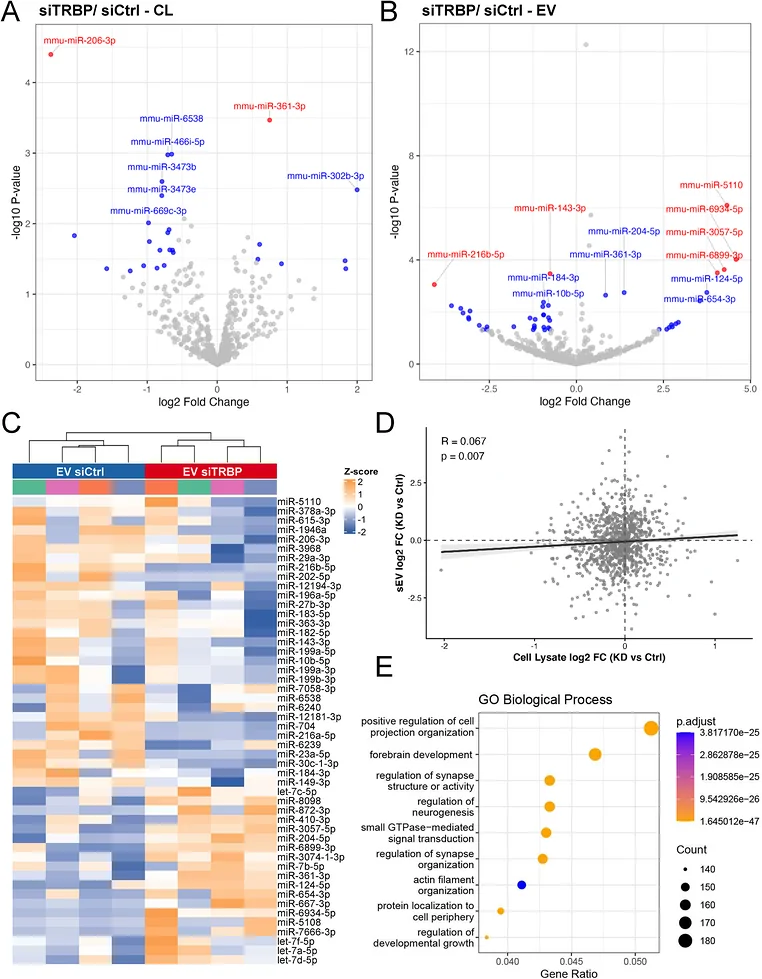
TRBP bi‐directionally affects miRNA abundance in neuronal EVs. (A) Volcano plot showing differentially expressed miRNAs in EVs from siCtrl and siTRBP‐treated neurons based on small RNA sequencing (*n* = 4). Red dots represent miRNAs with p.adj<0.1 and log(fold‐change)>0.58, and blue dots represent miRNAs with p<0.05 and log(fold‐change)>0.58. (B) Volcano plot of differentially expressed miRNAs between control and TRBP KD CL (n = 4). Red dots are miRNAs that changed significantly, p.adj<0.1 and |logFC|>0.58, and blue dots represent changes of p<0.05 and |logFC|>0.58. (C) Heatmap demonstrating the abundance of differentially expressed miRNAs in EVs. D) Correlation of fold‐changes in EV and CL miRNAs upon TRBP KD. (E) Gene ontology (GO) analysis of the predicted targets of differentially expressed miRNAs in siTRBP EVs compared to siCtrl.

Previous publications indicate the enrichment of RNA motifs in EV‐secreted and cell‐retained miRNAs [7, 11, 12]. We first examined the potential enrichment of 4–7mer motifs for all miRNAs in neuronal EVs compared to CL (Figure S3A). Although several putative motifs were significantly over‐represented in EV‐miRNAs, these did not represent a high percentage (< 6%) of the miRNA target population. We did however observe some long motifs containing previously annotated ‘core’ motifs for RBPs such as hnRNPA2B1 (*GGAG*), Annexin A2 (*AA[C/G][A/U]G*), and Lin28 (*GGAG[G/A]*), which reportedly play a role in EV‐miRNA secretion [23]. In contrast, motif enrichment analysis of miRNAs that were significantly regulated by TRBP KD in each fraction revealed significant enrichment of 6–7mer motifs (> 7.5% of targets, > 3‐fold enrichment compared to total; Figure 3A,B). Interestingly, the previously reported Syncrip‐binding motif *GGC[A/U]* appeared as the top enriched 6‐mer motif in EVs (19% of targets, 4.6‐fold enrichment; Figure 3A), but not in CL, suggesting that TRBP likely regulates the sorting of miRNAs with this ‘Syncrip‐like’ motif. We further investigated the putative functions of TRBP‐regulated miRNAs containing the core Syncrip motif, using synapse gene ontology analysis (*SynGO*) [24] of their predicted targets. Here, we detected a highly significant enrichment for neuronal synapse localization and function (Figure 3C and Tables S4 and S5), suggesting that the TRBP‐mediated sorting of Syncrip‐binding miRNAs could be important for synapse physiology. Apart from the Syncrip‐like motif, we detected the enrichment of 6–7mer motifs containing previously annotated sequences for EV‐RBPs such as hnRNPU (*CAUUG*, top third motif), La protein (*UUU* or *UGGA*, top third motif) and Annexin A2 (*AA[C/G][A/U]G*, top fourth motif) [23] in TRBP‐regulated EV miRNAs. Overall, this suggests that TRBP may regulate EV‐miRNA sorting via interactions with several EV‐RBPs, including Syncrip.

**FIGURE 3:**
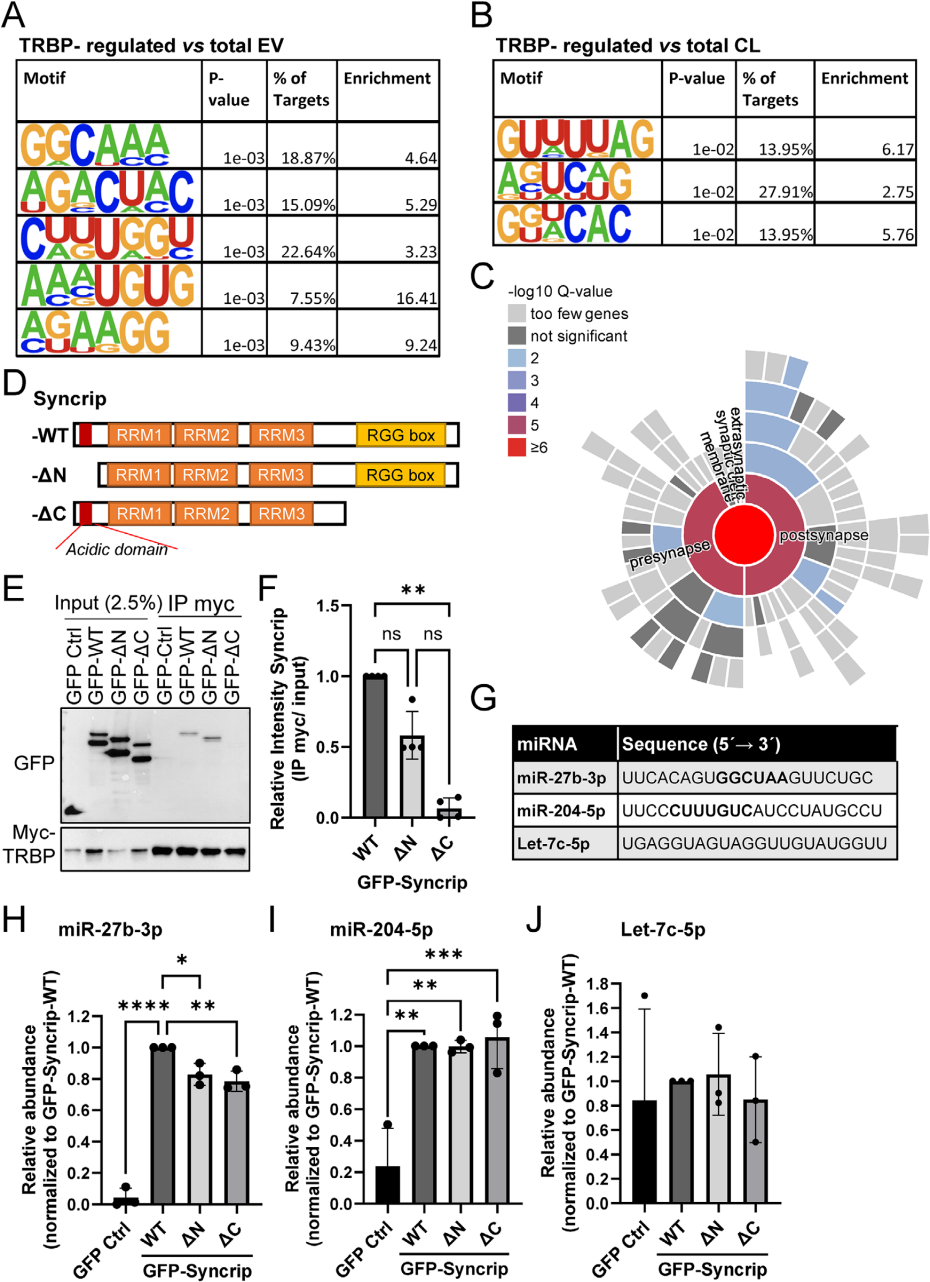
A TRBP binding‐deficient mutant of Syncrip shows decreased binding to TRBP‐regulated miRNAs with a Syncrip‐like motif. (A,B) Motif enrichment analysis for TRBP‐regulated miRNAs vs. all miRNAs in EVs (A) and CL (B). (C) Synaptic Gene Ontology (SynGO) analysis of differentially expressed EV‐miRNAs consisting of a Syncrip‐like motif. (D) Primary sequence of Syncrip‐WT, ‐∆N and ‐∆C, RRM; RNA recognition motif. (E) TRBP does not interact with the Syncrip‐∆C deletion mutant. N2a cells were co‐transfected with myc‐TRBP and GFP, GFP‐Syncrip‐WT, ‐∆N or ‐∆C, and IP was performed using an anti‐myc antibody. (F) Analysis of the relative intensity of Syncrip in IP as in E. IP intensity was normalized to corresponding input lysates, n = 4, **p<0.005, Kruskal Wallis test. (G) Table depicting three candidate mature miRNAs containing a Syncrip‐like motif (miR‐27b), another TRBP‐regulated EV motif (miR‐204) and no motif (let‐7c). Motif sequences are shown in bold letters. (H–J) RNA IP of candidate miRNAs using anti‐GFP antibody in N2a cells expressing GFP, GFP‐Syncrip‐WT, ‐∆N or ‐∆C, n = 3, *p = 0.02, **p<0.01, **p = 0.001, ***p<0.0001; 1way ANOVA.

### 2.3 The C‐Terminal Region of Syncrip Regulates its Interaction With TRBP and miRNAs With a Syncrip‐Like Motif

To gain mechanistic insight into the TRBP‐Syncrip interaction, we first performed protein interaction analysis using Syncrip deletion mutants. While deletion of the N‐terminal domain of Syncrip (GFP‐Syncrip‐ΔN), which was previously proposed to mediate miRNA binding [11], did not affect its interaction with myc‐tagged TRBP, deletion of the Syncrip C‐terminal domain (GFP‐Syncrip‐ΔC) abolished the interaction in N2a cells, compared to GFP‐Syncrip wild‐type (WT) (Figure 3D–F). Interestingly, the opposite was observed for the TRBP orthologue PACT, which was previously shown to be an RLC cofactor preferentially mediating the cleavage of pre‐siRNAs [25]. Here, we observed increased binding of endogenous PACT to GFP‐Syncrip‐ΔC, compared to ‐WT and ‐ΔN in IP (Figure S3B,C). This may indicate an alternative regulatory mechanism for the sorting of siRNAs, although further work would be necessary to verify this hypothesis. Consistently with IP experiments, we observed decreased co‐localization between mCherry‐TRBP and GFP‐Syncrip‐ΔC in N2a cells, compared to GFP‐Syncrip‐WT, and ‐ΔN (Figure S3D,E).

We next investigated whether the Syncrip TRBP‐interacting C‐terminal region is important for binding of miRNAs containing the Syncrip‐like EV motif, using RNA‐IP experiments in N2a cell lysates expressing GFP Ctrl, GFP‐Syncrip‐WT, ‐∆N or ‐∆C. Here, we detected the enrichment of the Syncrip‐like motif‐containing miRNA, miR‐27b‐3p, in GFP‐Syncrip‐WT IP compared to GFP Ctrl, which was significantly decreased in GFP‐Syncrip‐∆C as well as ‐∆N IPs (Figure 3G,H). In contrast, GFP‐Syncrip‐WT, ‐∆C, and ‐∆N IPs were equally abundant in miR‐204‐5p, which contains another predicted EV motif for TRBP‐regulated miRNAs, *CUUUGUC*, compared to GFP Ctrl (Figure 3G,I). Moreover, the miRNA let‐7c, which was regulated upon TRBP KD but does not contain any predicted motif sequences, did not interact with GFP‐Syncrip WT or deletion mutants (Figure 3G,J). Importantly, expression of GFP‐Syncrip‐WT, ‐∆C, or ‐∆N did not change the intracellular abundance of these miRNAs compared to GFP Ctrl (Figure S3F–H), or the total RNA concentration in small EVs isolated by SEC‐UC (Figure S3I). Taken together, this data supports the hypothesis that TRBP preferentially loads miRNAs containing a Syncrip‐like motif to Syncrip.

### 2.4 The TRBP‐Interacting Domain of Syncrip Mediates its Interaction With Ago2 in RNA Granules

TRBP is known to be important for the loading of mature miRNA duplexes to Ago proteins, the major catalysts of miRNA‐mediated translational repression by the RISC. As previous pull‐down experiments suggested that Syncrip may be a component of the RISC [26], we reasoned that TRBP may mediate interactions between Syncrip and Ago proteins. Indeed, we observed the interaction between HA‐tagged Ago2 and GFP‐Syncrip‐WT and ‐∆N, but not with the TRBP interaction‐deficient mutant, Syncrip‐∆C, in IP experiments in N2a cells (Figure 4A and Figure S4A). Expression of GFP‐Syncrip WT or deletion mutants did not influence the formation of the RLC, as we did not detect any changes in the interaction between HA‐Ago2 and endogenous Dicer (Figure S4B). We further observed the formation of punctate structures by GFP‐Syncrip‐WT and GFP‐Syncrip‐∆N, resembling RNA granules (Figure 4B and Figure S4C), which is in agreement with a previous study showing that Syncrip is present in granules containing YBX‐1 [8]. Co‐localization analysis revealed that approximately 30% of the total mCherry‐Syncrip‐WT and ‐∆N puncta overlap with GFP‐Ago2 and GFP‐Dcp1a (Figure 4C–E and Figure S4D), suggesting their presence in cytoplasmic P‐bodies, which are sites for miRNA‐mediated translational repression. In contrast, the C‐terminal deletion mutant of Syncrip had a more cytoplasmic and nuclear distribution, rarely forming punctate structures resembling RNA granules (Figure 4B–E and Figure S4C,D). Interestingly, overexpression of either mCherry‐Syncrip‐∆N or ‐∆C led to an increase in the number of GFP‐Ago2, but not GFP‐Dcp1a puncta compared to GFP‐Syncrip‐WT (Figure 4F,G), suggesting increased sorting of Ago2 in Dcp1a‐containing P‐bodies. Therefore, decreased binding of Syncrip to miRNAs or the TRBP‐containing RLC may lead to increased sorting of Ago2 to P‐bodies, likely caused by increased intracellular miRNA‐mediated translational repression [27].

**FIGURE 4:**
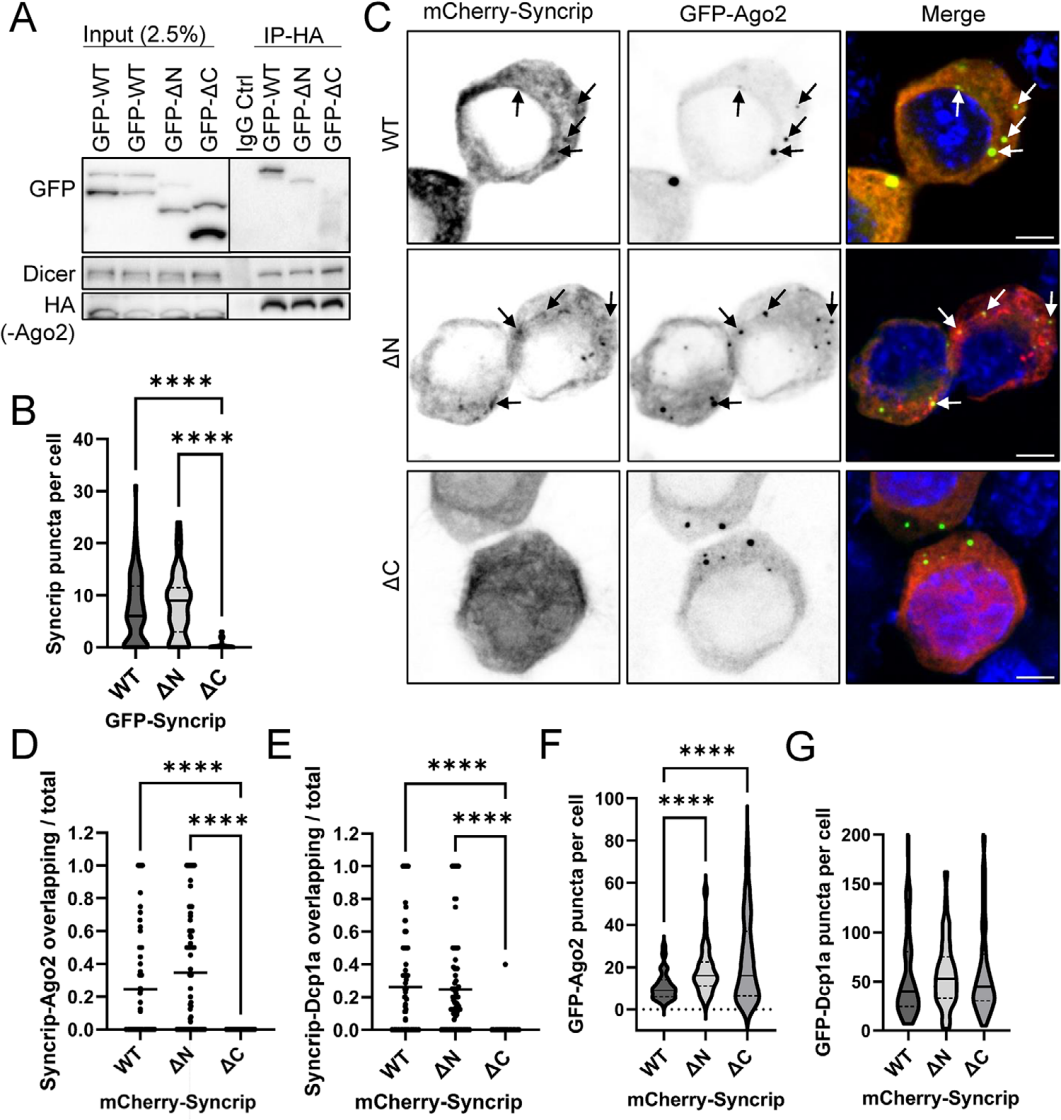
The C‐terminal TRBP‐binding domain of Syncrip mediates its interaction with Ago2 and localization to P‐bodies. (A) HA‐Ago2 binds to GFP‐Syncrip‐WT and ‐∆N, but not ‐∆C. IP was performed in N2a cells using anti‐HA antibody. (B) Quantification of Syncrip‐containing puncta resembling RNA granules, n = 50–90 cells, ****p<0.0001, 1way ANOVA. (C) Syncrip granules overlap with Ago2‐containing RNA granules. N2a cells were transfected with mCherry‐Syncrip‐WT, ‐∆N or ‐∆C, and GFP‐Ago2. Arrows point to overlapping puncta, DAPI shown in blue in merged images, scale bar; 5 µm. (D) Percentage of mCherry‐Syncrip puncta overlapping with GFP‐Ago2 granules. Line represents the mean, n = 47–90 cells from 3 independent experiments, ****p<0.0001, Kruskal‐Wallis test. (E) Percentage of mCherry‐Syncrip puncta overlapping with the P‐body marker, GFP‐Dcp1a. Line represents the mean, n = 77–98 cells from 3 independent experiments, ****p<0.0001, Kruskal‐Wallis test. (F) Expression of mCherry‐Syncrip‐∆N and ‐∆C increases the number of GFP‐Ago2 puncta compared to WT in N2a cells; 85–90 cells from 3 independent experiments, ****p<0.0001, 1way ANOVA. (G) There are no differences in the number of GFP‐Dcp1a puncta in N2a cells expressing mCherry‐Syncrip‐WT, ‐∆N or ‐∆C, n = 77–98 cells from 3 independent experiments.

### 2.5 ER‐MCS Regulate the Binding of Syncrip to ER‐Associated TRBP‐RLC and Secretion in EVs

We and others have shown that the TRBP‐RLC nucleates at the cytoplasmic surface of the ER [20, 21]. As dynamic interactions between the ER and endosomes were previously shown to regulate EV secretion [13, 28], we investigated whether ER‐endosome MCS may be important for the regulation of Syncrip‐RLC complex formation. First, we observed that GFP‐Syncrip puncta were in close proximity to ER tubules labeled with mCherry‐Climp63 in N2a cells (Figure S5A). Co‐localization analysis indeed shows decreased Mander's co‐efficients between GFP‐Syncrip‐∆C and mCherry‐Clim63, compared to GFP‐Syncrip‐WT and ‐∆N (Figure S5A,B). To examine the role of ER‐MCS, we transfected primary hippocampal neurons with GFP‐Protrudin WT, which forms ER‐MCS, or the mutant FYVE4A, which does not form ER MCS [29]. Expression of GFP‐Protrudin‐FYVE4A led to an increase in the co‐localization of endogenous Syncrip with mCherry‐TRBP and GFP‐Protrudin in hippocampal neuron dendrites (Figure 5A–C), suggesting that inhibition of ER‐MCS increase the recruitment of Syncrip to ER‐associated TRBP. Proximity ligation assay (PLA) confirmed the increased interaction between endogenous TRBP and Syncrip in hippocampal neurons expressing GFP‐Protrudin‐FYVE4A compared to WT (Figure 5D,E). Furthermore, we observed decreased co‐localization between Syncrip and Rab7‐labeled late endosomes in hippocampal neuron dendrites expressing GFP‐Protrudin‐FYVE4A, compared to GFP Ctrl and GFP‐Protrudin‐WT (Figure 5F and Figure S5C). As expected, GFP‐Protrudin‐FYVE4A also displayed decreased co‐localization with Rab7 (Figure 5G), verifying decreased interactions between the ER and late endosomes, compared to Protrudin‐WT. Interestingly, expression of GFP‐Protrudin‐FYVE4A in N2a cells also led to the increased abundance of Syncrip and Ago (*p* = 0.09) in EVs isolated by IP using CD63/‐81/‐9 antibody‐coated beads, compared to GFP Ctrl and GFP‐Protrudin WT (Figure 5H–J). Overall, this data suggests that inhibition of ER‐MCS induces the recruitment of Syncrip to ER‐associated TRBP‐RLC, likely from Rab7‐positive endosomes, thereby promoting the sorting of Syncrip and Ago to EVs.

**FIGURE 5:**
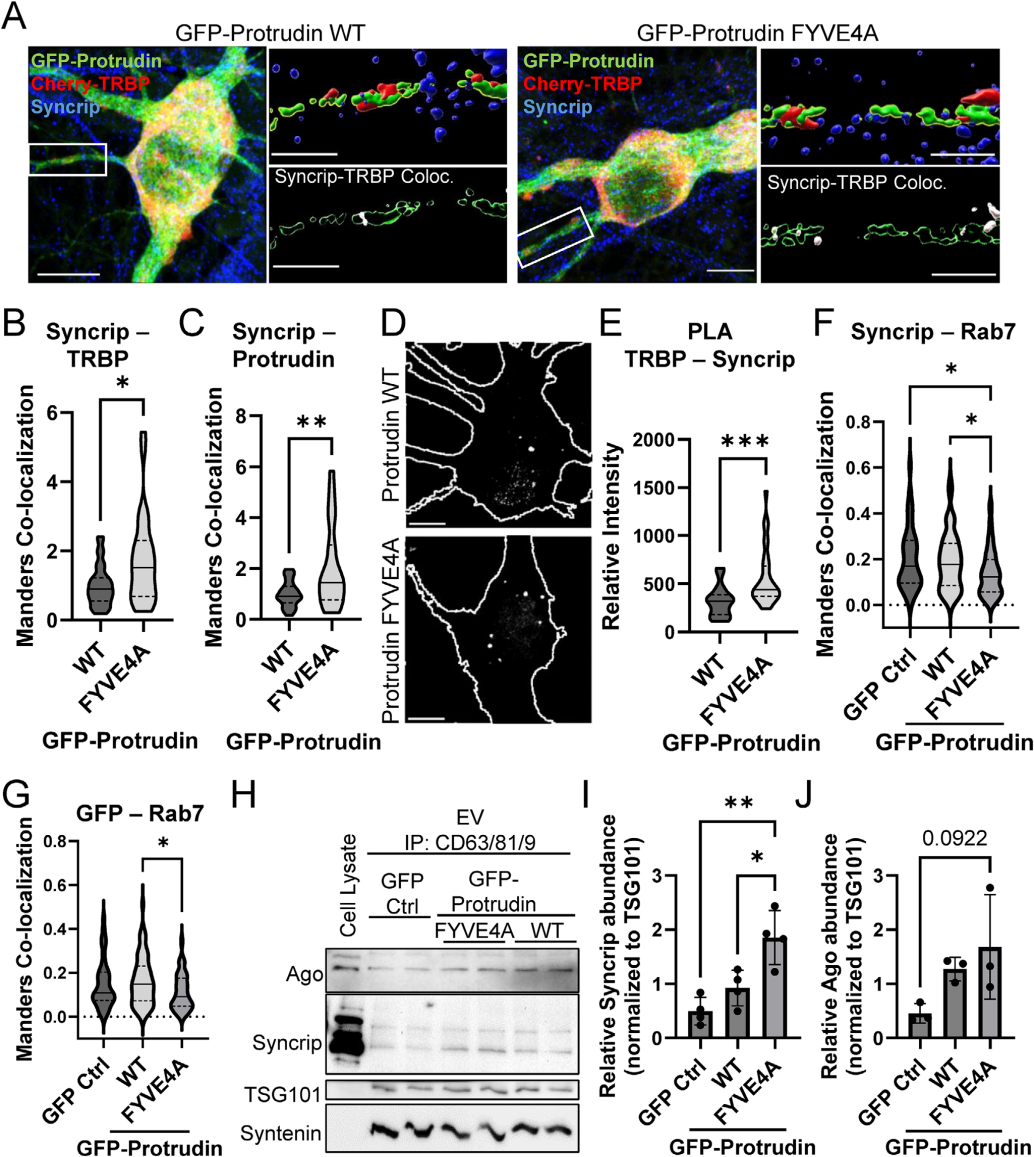
ER‐MCS regulate the interaction between Syncrip and TRBP. (A) Airyscan images of a 7DIV hippocampal neuron dendrite expressing GFP‐Protrudin‐WT or ‐FYVE4A and mCherry‐TRBP and immunostained with Syncrip antibodies. Segmentation in dendrites is shown for boxed ROIs; mCherry‐TRBP; red, Protrudin; green, Syncrip; blue, Syncrip‐TRBP co–localization; white, scale bar; 5 µm. (B) Inhibition of ER MCS increases TRBP‐Syncrip co‐localization. Co‐localization analysis was performed for images acquired as in A, n = 30, *p = 0.01, Welsch t‐test. (C) Inhibition of MCS increases Syncrip localization to the ER. Co‐localization analysis between Syncrip and Protrudin in images acquired as in A, n = 30, **p = 0.002, Welsch t‐test. (D) Proximity ligation assay in primary hippocampal neurons. GFP was used as a morphological mask, indicated by the white overlay, scale bar: 5 µm. (E) Analysis of PLA signal in images as in D, n = 25‐29, ****p<0.0001, ***p = 0.0005, 1way ANOVA. (F) Manders co‐localization co‐efficients of mCherry‐Syncrip and Rab7 in neuronal dendrites expressing GFP Ctrl, GFP‐Protrudin‐WT or ‐FYVE4A, n = 46–71, *p<0.03, 1way ANOVA. (G) Co‐localization analysis between Rab7 and GFP, GFP‐protrudin‐WT or ‐FYVE4A, n = 46‐72; *p = 0.01, 1way ANOVA. (H) Immunoprecipitation of EVs using CD63/‐81/‐9 antibody coated beads. Donor N2a cells were transfected with GFP Ctrl, GFP‐Protrudin WT, or FYVE4A as depicted. (I) Relative abundance of Syncrip in EV‐IP as in H. Values were normalized to TSG101, n = 4, *p = 0.01, **p = 0.001, 1way ANOVA. (J) Relative abundance of Ago in EV‐IP as in H. Values were normalized to TSG101, n = 4, 1way ANOVA.

### 2.6 Endosome‐MCS Regulate the Localization of Syncrip and Ago2

To verify that ER‐MCS with endosomes regulate Syncrip‐TRBP co‐localization, we performed experiments in primary neurons expressing the endosomal protein mCherry‐ORP1L‐WT, ‐∆ORD, which increases the formation of endosome‐MCS, or ‐∆ORDPHDPHD, which does not form endosome‐MCS [29]. Consistently with the above findings using Protrudin, increased endosome‐MCS formation via expression of mCherry‐ORP1L‐∆ORD decreased the co‐localization between Syncrip and GFP‐TRBP, compared to mCherry‐ORP1L‐WT or ‐∆ORDPHDPHD (Figure 6A,B). In contrast, we did not observe any changes in the co‐localization between GFP‐PACT and Syncrip under the same experimental conditions (Figure 6C and Figure S6A).

**FIGURE 6:**
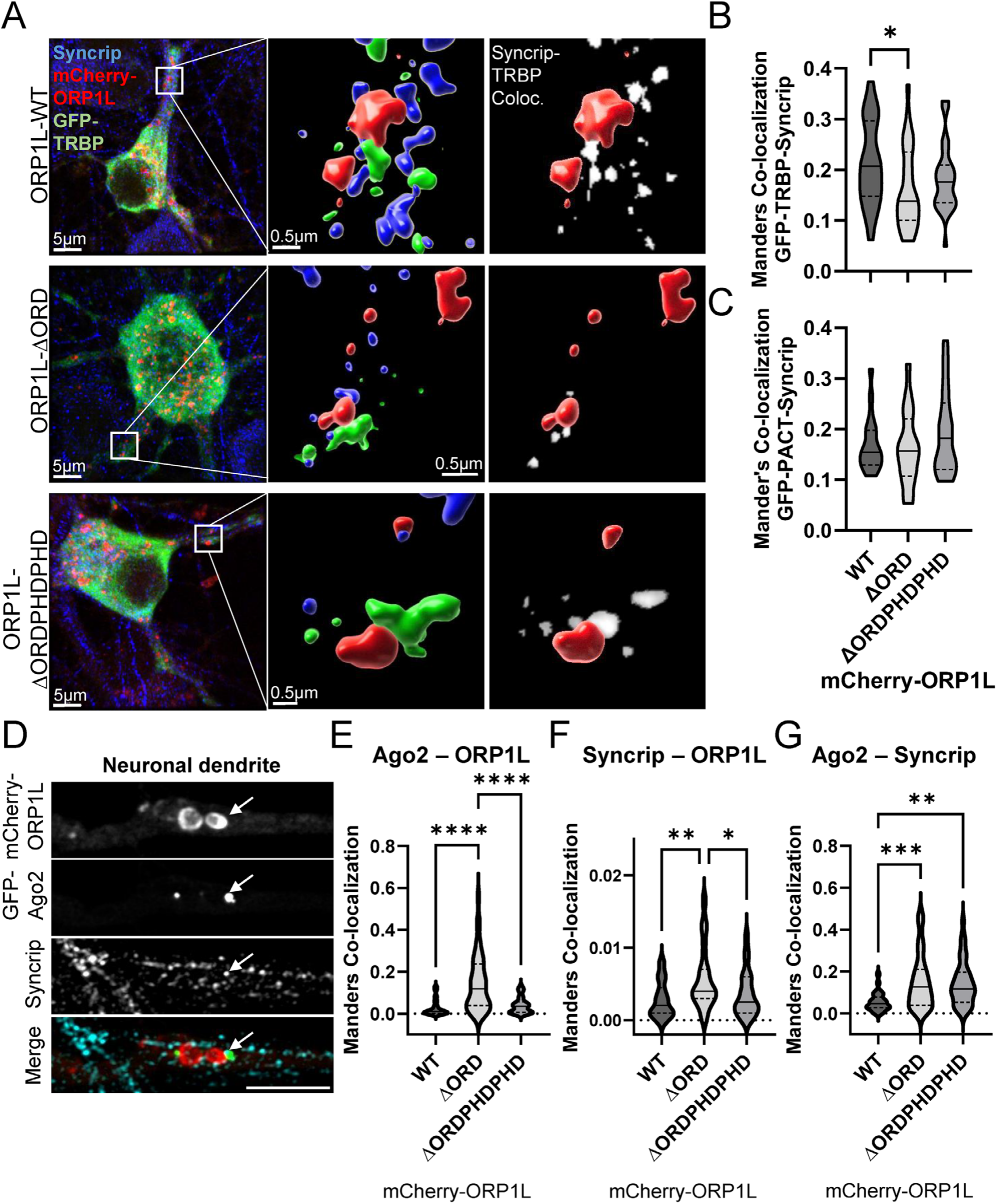
Endosome MCS regulate the localization of Syncrip and Ago2 to endosomes. (A) Airyscan images of 7DIV primary hippocampal neurons expressing mCherry‐ORP1L‐WT, ‐∆ORD or ‐∆ORDPHDPHD and GFP‐TRBP, and immunostained with Syncrip antibodies. Segmentation of selected ROI is shown, cherry‐ORP1L; red, GFP‐TRBP; green, Syncrip; blue, Syncrip‐TRBP co‐localization; white. (B) GFP‐TRBP and Syncrip co‐localization analysis in images acquired as in A, n = 18‐30, *p = 0.04, 1way ANOVA. (C) GFP‐PACT and Syncrip co‐localization analysis of Airyscan images of primary neurons expressing mCherry‐ORP1L‐WT, ‐∆ORD or ‐∆ORDPHDPHD, and GFP‐PACT, and immunostained with Syncrip antibodies, n = 18‐29. (D) Airyscan images of a 7DIV hippocampal neuron dendrite expressing mCherry‐ORP1L and GFP‐Ago2 and immunostained with Syncrip antibody. Arrow points to co‐localizing Ago2‐Syncrip puncta on the surface of ORP1L‐positive endosomes, scale bar: 5 µm. (E–G) Manders co‐localization coefficients between Ago2 (E) or Syncrip (F) with ORP1L, and between Ago2 and Syncrip (G) in dendrites of primary hippocampal neurons, n = 32‐48, *p = 0.01, **p<0.01, ***p<0.001, ****p<0.0001.

Given previous reports indicating the association of RNA granules with endosomes in neurites [30], and our observation that a fraction of Syncrip is present in P‐bodies (Figure 4C–E and Figure S4D), we next examined whether endosome‐MCS influence the association of Syncrip with Ago2‐positive RNA granules. Here, we observed the co‐localization of GFP‐Ago2 granules on the surface of mCherry‐ORP1L labeled endosomes, which also consisted of Syncrip (Figure 6D). Interestingly, both GFP‐Ago2‐positive granules and Syncrip puncta displayed increased co‐localization with mCherry‐ORP1L‐∆ORD, compared to mCherry‐ORP1L‐WT and mCherry‐ORP1L‐∆ORD‐PHDPHD (Figure 6E,F and Figure S6B). Moreover, expression of either mCherry‐ORP1L‐∆ORD, or ‐∆ORD‐PHDPHD caused an increase in the co‐localization of Syncrip to GFP‐Ago2 puncta resembling P‐bodies (Figure 6G and Figure S6B). This suggests that the incorporation of Syncrip to Ago2‐positive granules is dynamically regulated by endosome‐MCS, with increased formation of ER‐endosome MCS promoting the recruitment of Syncrip and Ago2 granules to endosomes (Figure S6C).

### 2.7 Syncrip Binding to TRBP is Important for the Differential Regulation of Synapse Formation in EV Donor and Recipient Neurons

We next explored the potential functional significance of this pathway in neuronal dendrite and synapse formation. Overexpression of GFP‐Syncrip‐WT, ‐ΔC or ‐ΔN did not significantly alter the complexity in 10DIV hippocampal neuron dendrites (‘donor neurons’) compared to GFP Ctrl, as assessed by Sholl analysis (Figure 7A–C). In addition, SEC‐UC‐isolated EVs derived from neurons transduced with AAVs expressing GFP‐Syncrip‐WT, ‐ΔC or ‐ΔN did not affect dendrite complexity in recipient hippocampal neurons compared to EVs derived from non‐transduced neurons (Figure 7D,E and Figure S7A). Importantly, overexpression of GFP‐Syncrip‐WT, ‐ΔC or ‐ΔN did not significantly change EV size or concentration as assessed by nanoparticle tracking analysis (Figure S7B–D).

**FIGURE 7:**
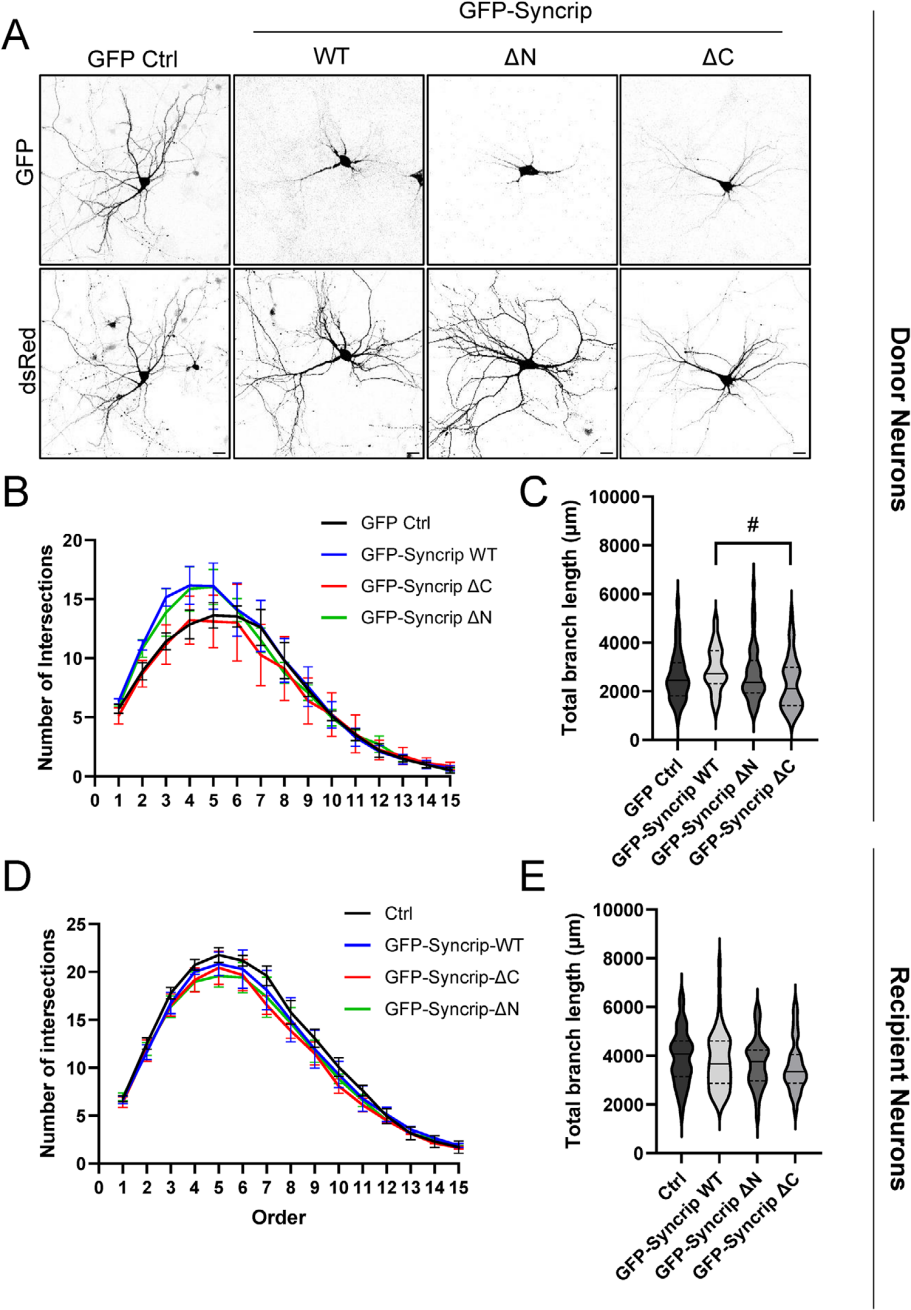
Expression of GFP‐Syncrip‐WT, ‐∆N, or ‐∆C does not influence dendrite arborization in donor or recipient neurons. (A) Primary neurons were co‐transfected with dsRed and either GFP Ctrl, GFP‐Syncrip‐WT, ‐∆C or ‐∆N at 5–10 DIV. Shown are inverted confocal images, scale bar; 20 µm. (B) Sholl analysis profiles of dsRed expressing neurons as shown in A. Approximately 10 neurons were analyzed in each of 4 independent experiments. Error bars represent standard error of the mean, n = 4. (C) Total branch length of neurons treated as in A, n = 32‐48, #*p* = 0.08, 1way ANOVA. (D,E) Sholl profiles (D) and total branch length (E) of recipient neurons treated with EVs from neurons transduced with GFP‐Syncrip‐WT, ‐∆C, ‐∆N or EV Ctrl in 4 independent experiments as in B&C, n = 50‐63, 1way ANOVA.

To investigate whether the TRBP‐mediated loading of miRNA to Syncrip regulates synapse formation, we analyzed synapse density in donor neurons expressing GFP‐Syncrip‐WT, ‐ΔC or ‐ΔN, and in recipient neurons treated with small EVs isolated by SEC‐UC from GFP‐Syncrip‐WT, ‐ΔC or ‐ΔN‐expressing neurons, as above. Donor and recipient hippocampal neurons were immunostained with antibodies against the pre‐synaptic protein Synapsin and the post‐synaptic protein Homer, and synapse density was analyzed by quantifying the abundance of Synapsin puncta or Synapsin‐Homer co‐localization in proximity to neuronal dendrites expressing GFP (donor) or dsRed (recipient). In donor neurons, GFP‐Syncrip‐WT, but not ‐ΔN or ‐ΔC overexpression led to an increase in Synapsin‐Homer co‐localization and the number of Synapsin puncta compared to GFP Ctrl (Figure 8A–C and Figure S8A). On the contrary, recipient neurons treated with EVs from neurons transduced with AAV‐GFP‐Syncrip‐ΔN or ‐ΔC displayed increased synapse density compared to EV Ctrl and AAV‐GFP‐Syncrip‐WT (Figure 8D–F and Figure S8B). To investigate whether the RNA content of EVs is responsible for observed phenotypes in recipient neurons, we depleted RNA from EVs using RNase A treatment in the presence of the mild detergent Saponin, prior to SEC‐UC and incubation with hippocampal neurons. Saponin has been extensively used to load EVs with selected protein cargo, as it reversibly allows permeability of the EV membrane without influencing overall EV properties [31]. We first verified that RNase A treatment of EVs in the presence of Saponin reduces the RNA concentration of SEC‐UC‐isolated EVs (Figure S8C). Depletion of EV‐RNA cargo blocked the observed increase in Synapsin‐Homer co‐localization in recipient dendrites treated with EVs from GFP‐Syncrip‐ΔC‐expressing neurons (Figure 8G and Figure S8D), thereby indicating the importance of EV‐RNA cargo. Interestingly, this was not the case for RNA‐depleted EVs isolated from GFP‐Syncrip‐ΔN‐expressing neurons, which were still able to increase synapse density in recipient neurons (Figure 8G and Figure S8D). To further investigate the potential role of the non‐vesicular extracellular compartment, we also performed experiments using the EV‐depleted protein fraction isolated by SEC‐UC. Here, EV‐depleted fractions derived from GFP‐Syncrip‐ΔN (but not ‐ΔC)‐expressing neurons decreased synapse density compared to GFP‐Syncrip‐WT (Figure 8H and Figure S8E). We therefore speculate that while the interaction between Syncrip and TRBP via the Syncrip C‐terminus modulates synapse formation via EV‐associated miRNAs, the Syncrip N‐terminal region may likely influence synapse formation through additional changes in the protein composition of EVs, as well as non‐vesicular fractions.

**FIGURE 8:**
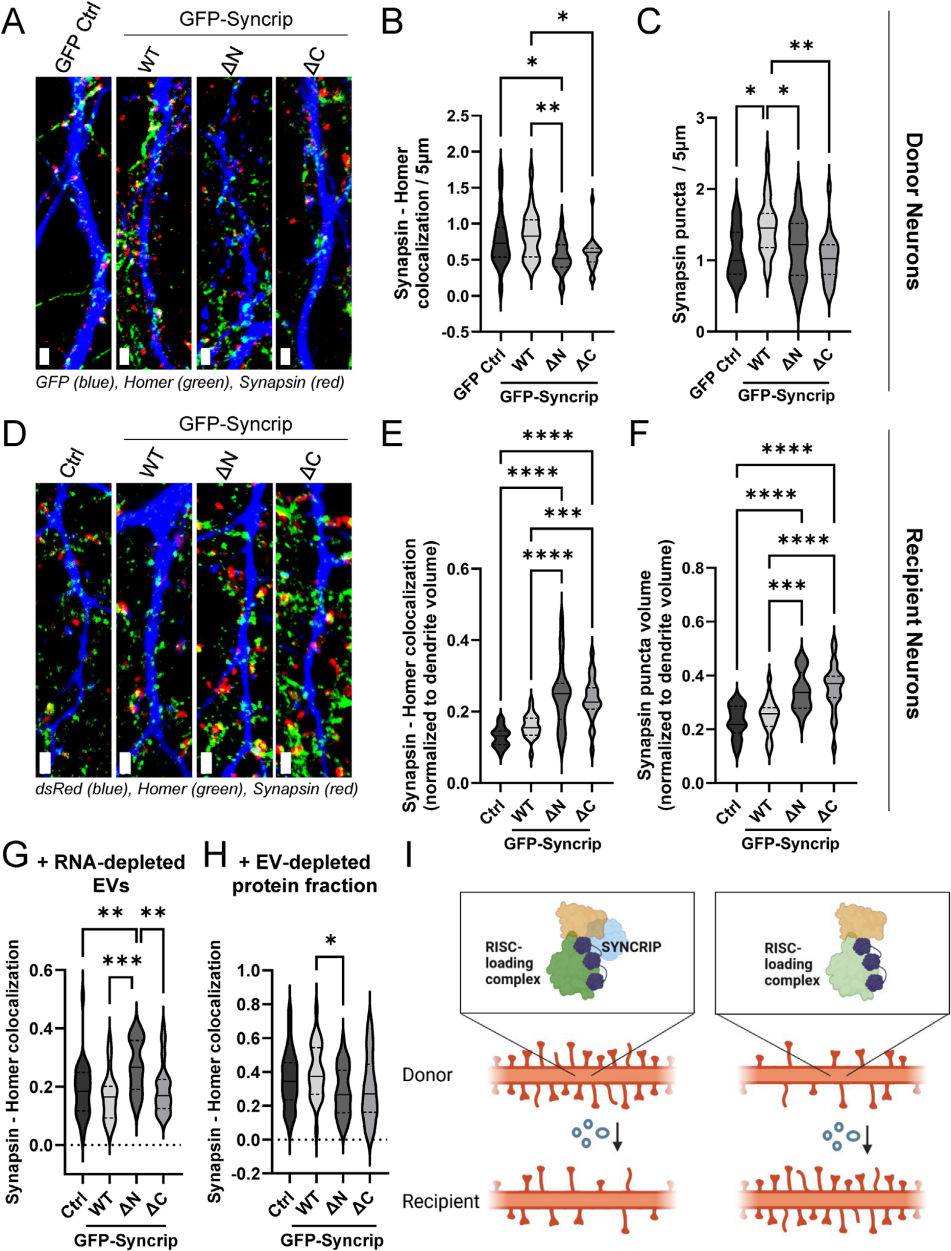
Differential regulation of synapse formation in donor and recipient neurons by Syncrip WT and mutant over‐expression. (A) Primary neurons were transfected with GFP‐Ctrl, GFP‐Syncrip‐WT, ‐∆C or ‐∆N and immunostained with Synapsin and Homer antibodies at 10DIV. Scale bar 2 µm. (B,C) Overexpression of GFP‐Syncrip‐∆C or ‐∆N decreases the number of synapses in donor neurons. The number of Synapsin‐Homer co‐localizing puncta (B) and the number of Synapsin puncta within 2 µm from dendrites (C) were quantified in dendrites treated as in A and normalized to dendrite length in 3 independent experiments, n = 20–23, *p< 0.05, **p<0.005, 1way ANOVA. (D) Primary neurons were treated with EVs from donor neurons transduced with GFP‐Syncrip‐WT, ‐∆C, ‐∆N or EV Ctrl and stained with Synapsin and Homer antibodies. Scale bar 2 µm. (F,G) EVs from neurons overexpressed GFP‐Syncrip‐∆C or ‐∆N increased the synapse numbers in recipient neurons as in D. The Synapsin‐Homer co‐localizing puncta (E) and the Synapsin puncta (F) within 2 µm from dendrites were normalised to the dendrite volume (dsRed labeled). 3 independent experiments, n = 19‐22. ***p = 0.0005, ****p<0.0001, 1way ANOVA. (G) EVs from donor neurons were treated with Saponin and RNase A, and then applied to recipient neurons. Synapse‐Homer co‐localization in 50µm‐long dendrites was quantified in 2 independent experiments, n = 29‐31, ***p = 0.0001, **p<0.01, 1way ANOVA. (H) Recipient neurons were treated with protein fractions isolated by SEC and concentrated by UC as depicted in 3 independent experiments, n = 29‐54, *p = 0.02, 1way ANOVA. (I) Schematic depicting proposed model. Created in BioRender (Antoniou, A. (2026) https://BioRender.com/z4quwkg).

Taken together, these results indicate that Syncrip differentially regulates synapse formation in donor and recipient neurons, which is dependent on TRBP binding and EV‐RNA cargo. Overall, we demonstrate that the incorporation of Syncrip at ER‐associated TRBP favors synapse formation in donor neurons, whereas inhibition of the TRBP‐Syncrip interaction promotes synapse formation in recipient neurons (Figure 8I). The local regulation of Syncrip binding to the ER‐associated TRBP in neuronal dendrites could be important for the fine‐tuning of donor and recipient synapses and thus regulate neuronal circuit formation during development.

## 3 Discussion

Although several RBPs were shown to play a role in RNA sorting to EVs, the precise molecular mechanisms are still largely unclear. This study demonstrates that the ER‐associated TRBP‐containing RLC acts as a ‘hub’ for miRNA sorting, through interactions with Syncrip, and likely other RBPs. The dynamic formation of ER‐endosome MCS further regulates the localization of Syncrip to late endosomes, Ago2‐containing RNA granules, as well as EVs. This pathway could in turn modulate the local sorting and secretion of functional miRNA complexes from neuronal dendrites. Indeed, expression of a TRBP‐binding‐deficient mutant of Syncrip increased the incorporation of Ago2 in P‐bodies and decreased synapse formation in donor neurons, while EVs from these neurons increased synapse formation in recipient neurons. We thereby speculate that ER‐associated TRBP may function as a gatekeeper for retaining synaptic miRNAs, where dynamic changes in RBP recruitment and loading with miRNA regulates EV‐miRNA composition and in turn fine‐tunes synapse formation in recipient neurons. Importantly, this knowledge not only advances our understanding of miRNA‐sorting pathways, it could benefit the development of novel therapeutic approaches via the engineering of EVs for RNA delivery.

### 3.1 The TRBP‐Containing RLC as a Hub for miRNA Sorting

TRBP is a major component of the RLC, and was shown to be important for the accuracy of pre‐miRNA cleavage by Dicer and the loading of the cleaved miRNA to Ago proteins [16, 17, 18]. Importantly, we did not observe a correlation between TRBP‐dependent changes in the abundance of miRNAs in neuronal cell lysates and corresponding EVs, suggesting specific sorting mechanisms for each compartment. However, this data does not exclude indirect effects of impaired pre‐miRNA cleavage and/or RISC loading on EV‐miRNA abundance. Nevertheless, our mass spectrometry data of endogenous TRBP IP suggests that TRBP interacts with most of the RBPs previously implicated in the sorting of miRNAs to EVs, including Syncrip, YBX‐1 and hnRNPA2B1. Moreover, we observed the enrichment of 6–7mer RNA motifs in EV miRNAs regulated by TRBP KD containing sequences for several previously reported RBPs, including Syncrip, hnRNPU and La protein, which were detected in neuronal EVs and TRBP IP by mass spectrometry. Notably, it is still unclear whether the binding of EV‐RBPs to RNAs is strictly determined by these motif stretches or whether other factors like secondary structure or RBP‐protein interactions are also important. Our results indeed suggest that additional factors likely mediate miRNA binding by Syncrip, as we detected the significant enrichment of a miRNA without a Syncrip motif, but with another putative EV motif, in Syncrip IP. This could also explain the relatively small albeit highly significant decrease in the binding of a miRNA containing the Syncrip‐like motif upon deletion of the N‐terminal region of Syncrip, previously shown to mediate miRNA binding [11]. The C‐terminal mutant of Syncrip that doesn't bind TRBP or Ago2 displayed a similar, significant decrease in the binding of this miRNA, suggesting that association with the miRNA production machinery is likely required for efficient binding to Syncrip‐motif containing miRNAs and thus their potential sorting to EVs. The relatively small changes in miRNA binding by the N‐ and C‐terminal mutants of Syncrip could also be explained by complex formation with other RBPs, such as PACT, which is known to form heterodimers with TRBP, and displayed increased binding to the C‐terminal mutant of Syncrip. Further high‐throughput investigations should be aimed at delineating the direct contribution of the RLC and associated RBPs, as well as EV motifs on neuron‐specific EV‐miRNA sorting.

Although we did not observe the clear enrichment of previously reported core ‘*CELL’* or ‘*EXO’* motifs in primary neurons, we detected the presence of common core motifs in EVs compared to donor neurons consisting of the hnRNPA2B1 recognition sequence, *GGAG* [23], as well as *UGUG*, *CAUG*, and *GAGG*, which were previously shown to be enriched in EVs from several cell types [12]. Apart from the Syncrip‐binding core motif and *UGUG*, these motifs were not particularly enriched in TRBP‐regulated EV‐miRNAs. Interestingly, a recent publication suggests that hnRNPA2B1, which is a known ‘reader’ of m6a RNA methylation [32], sorts m6a‐modified miRNAs in EVs that cannot associate with Ago2 [33], indicating that hnRNPA2B1‐associated EV‐miRNAs are likely not functional in recipient cells. Based on our observation that expression of Syncrip‐ΔC and ‐ΔN increases the incorporation of Ago2 to RNA granules, presumably as a consequence of increased miRNA‐mediated translational repression [34], we speculate that unlike hnRNPA2B1, Syncrip may directly regulate Ago‐bound miRNAs through interactions with TRBP and the RLC. Future investigations should thus be directed to elucidate the precise roles of TRBP‐associated RBPs and/or putative EV motifs on the functionality of EV‐miRNAs in recipient cells.

### 3.2 ER‐Endosome MCS Dynamically Regulate Syncrip and Ago Localization and Sorting to EVs

We previously showed the existence of at least two populations of the RLC, containing TRBP and/or PACT or Dicer alone, and observed that TRBP‐RLC was primary localized at the cytoplasmic surface of the ER in primary neurons [20]. In addition, recent evidence in non‐neuronal cells suggests that ER‐MCS regulate the secretion of EVs and EV‐RNA in a dynamic manner [13, 28]. Our data shows that inhibition of ER‐endosome MCS increases the recruitment of Syncrip to the ER‐associated TRBP, and promotes the sorting of Syncrip, as well as Ago, to CD81/‐63/‐9‐positive EVs. On the other hand, induction of ER‐MCS, results in the re‐distribution of Syncrip to late endosomes in neuronal dendrites and similarly leads to a partial, albeit non‐significant increase in Syncrip and Ago sorting to EVs. These results could be explained by the presence of endogenous ER‐endosome MCS linker proteins, as also evidenced by the non‐significant increase in ER‐endosome co‐localization by expression of Protrudin‐WT compared to Ctrl, and the overall transient and localized nature of ER‐endosome MCS formation. Nevertheless, our results overall indicate a mechanism where Syncrip recruitment to the ER promotes its binding to the RLC and subsequent sorting to secretory vesicles, likely via the exosome biogenesis pathway.

Interestingly, we observed similar dynamics for Ago2‐positive RNA granules, where the induction of endosome‐MCS led to increased co‐localization between Ago2 granules and late endosomes. Consistent with this observation, Ago2 was previously shown to be present in endosomes in neuronal dendrites [35], and RNA granules were shown to ‘hitch‐hike’ with endosomes for long distance transport in axons [30]. As the ER extends into dendrites [36], and forms a platform for the nucleation of TRBP‐RLC [20], ER‐endosome MCS may thus be important for the sorting of *de novo* miRNAs in proximity to synapses. Importantly, impairment of ER‐endosome MCS has been implicated in several neurological disorders such as Amyotrophic Lateral Sclerosis and neurodegenerative diseases [37], in which EVs also play an important role [38]. This warrants further investigations of this pathway in disease‐relevant contexts.

### 3.3 Neuronal EVs May Function to Strengthen Micro‐Circuits During Development by Weakening Adjacent Synapses

We observed opposing effects in synapse density in donor and recipient neurons, upon overexpression of the miRNA‐ and TRBP‐binding deficient mutants, Syncrip‐ΔN, and ‐ΔC, respectively. Conversely, we did not observe any EV‐mediated changes in neuronal dendrite outgrowth, thus supporting a specific role for EV contents in synapse formation and arguing against general trophic support. While expression of Syncrip‐WT, but not Syncrip‐ΔN or ‐ΔC, increased synapse density in donor neurons, EVs from Syncrip‐ΔN or ‐ΔC expressing neurons, but not Syncrip‐WT, increased synapse density in recipient neurons. Notably, although EV‐RNA cargo depletion blocked the EV‐mediated induction in synapse formation by EVs from GFP‐Syncrip‐ΔC‐expressing donor neurons, this was not the case for EVs derived from GFP‐Syncrip‐ΔN‐expressing neurons. Together with the finding that EV‐depleted protein fractions from these neurons also influences synapse formation in recipient neurons, this data suggest that RNA cargo is not solely responsible for the observed synaptic phenotypes. We therefore speculate that the N‐terminal region of Syncrip may have additional roles apart from miRNA binding that influence the protein cargo of EVs, and potentially non‐vesicular extracellular fractions. Interestingly, it was previously shown that this region mediates protein interactions with APOBEC1, thus regulating the production and secretion of interleukin 6 [39]. Nevertheless, our data provide evidence that changes in EV‐RNA cargo downstream the TRBP‐Syncrip pathway are responsible for changes in synapse formation in recipient neurons.

SynGO analysis of the targets of TRBP‐regulated EV‐miRNAs that contain a Syncrip‐binding motif demonstrated enrichment of synaptic processes and functions. Among miRNAs containing a Syncrip‐like motif, miR‐27b‐3p, which was down‐regulated in EVs upon TRBP KD was previously shown to regulate the pre‐synaptic transcriptome [40]. Furthermore, TRBP‐regulated, Syncrip‐motif‐containing miRNAs, miR‐182‐5p, and miR‐183‐5p, were previously shown to belong to a cluster that regulates long‐term memory formation [41]. Our results therefore suggest that the TRBP‐mediated regulation of miRNA sorting via Syncrip favors the strengthening of donor neuron synapses, while the sorting of EV‐miRNAs with synaptic functions may be locally regulated through increased ER‐endosome MCS. A similar observation of synaptic competition via EVs was made with Arc‐containing EVs, which were shown to downregulate AMPA receptor endocytosis in recipient cells, thereby tuning synaptic strength in vitro [42]. Several publications have also indicated the EV‐mediated transfer of cargo proteins and RNA across synapses [6, 42, 43, 44, 45]. The fine‐tuning of synaptic strength may thus be a common feature of nervous system EVs, via the local regulation of neuronal processes.

### 3.4 Toward a Mechanistic Understanding of EV‐miRNA Signatures

Although several RBPs have been implicated in the sorting of EV‐miRNAs in various cell types, they are often studied in isolation. Our results suggest that the intracellular miRNA production machinery might be an important sorting ‘hub’ for dictating EV miRNA composition, and potentially miRNA cargo activity in recipient cells. Notably, we also observed the secretion of Ago2 in small EVs from primary neurons, implying that neuronal EVs are likely capable of delivering functional Ago‐miRNA complexes to recipient cells. In light of the recent controversies surrounding the secretion of functional Ago complexes in EVs [46], we speculate that EV‐miRNA delivery may be more relevant in contexts where local regulation of translation is important, such as the nervous system [47]. Furthermore, as miRNAs often target multiple, sometimes connected biological pathways, with mRNAs often consisting of multiple miRNA binding sites, we propose that the miRNA signature of ‘functional’ miRNA‐containing EVs, and how it is regulated by RBPs or other pathways, would be of more biological relevance than studying EV‐miRNAs in isolation. As EVs hold promise in drug delivery applications, this knowledge could ultimately aid EV engineering approaches for RNA‐based therapies.

## 4 Methods

### 4.1 Plasmids

Plasmids for GFP‐SYNCRIP, Flag/HA‐Ago2, and GFP‐Dcp1a were kindly provided by Gerhard Schratt. Plasmids for Protrudin and ORP1L were kindly provided by Frederik Verweij. mCherry‐Climp63 was kindly provided by Cyril Hanus. pAAV‐hSyn‐GFP was kindly provided by Sebastian Kuegler. GFP‐Ago2 was kindly provided by Phil Sharp (Addgene plasmid # 21981; RRID:Addgene_21981) [48]. TRBP was kindly provided by Dong‐Yan Jin (Addgene plasmid # 15666; RRID:Addgene_15666) [25]. hnRNPA2B1 was kindly provided by Nicolas Fawzi (Addgene plasmid # 98662; RRID:Addgene_98662) [49].

SYNCRIP deletion mutants were cloned using the Phusion Hot Start II DNA polymerase (Thermo Fisher) with GFP‐SYNCRIP as a template and ‐ΔC or ‐ΔN primers according to manufacturer's instructions. For mCherry‐Syncrip, mCherry was sub‐cloned into GFP‐Syncrip‐WT, ‐ΔN, and ‐ΔC after excision of GFP using *AgeI* and *XhoI*. For pAAV‐GFP‐Syncrip constructs, the sequences flanking Syncrip in GFP‐Syncrip‐WT, ‐ΔN, and ‐ΔC were amplified by PCR and sub‐cloned into pAAV‐hSyn‐Cherry‐CD81 using *NheI* and *HindIII*. Recombinant AAVs (AAV2/1) were produced at the viral core facility of Charité, Universitätsmedizin Berlin.

For myc‐tagged TRBP, a *HindIII* and *BamHI* flanked oligonucleotide was sub‐cloned in pcDNA‐TRBP. For myc‐hnRNPA2B1, hnRNPA2B1 was amplified by PCR using *BamHI*‐ and *XhoI*‐flanked oligonucleotides and subcloned into pcDNA3‐myc following excision of TRBP. mCherry‐TRBP was subcloned using *NheI* and *KpnI* by replacing GFP in GFP‐TRBP with mCherry excised from mCherry‐C1.

### 4.2 Cell Culture

Cells were cultured in humidified incubators with 5% CO_2_ at 37°C. Mouse neuroblastoma N2a cells (RRID:CVCL_0470) were cultured in DMEM medium (Gibco) with 2 mm Glutamax, 100U/mL Penicillin and 0.1 mg/mL Streptomycin and 10% fetal bovine serum (Capricorn). They were passaged every 2–3 days with 0.25% trypsin when the confluency reached 90%–100%. Cells within passage number 25 were used.

Primary cortical or hippocampal neurons were obtained from embryonic day 16–17 (E16‐17) NMRI mouse embryos according to local Animal Welfare regulations (LANUK NRW, 81‐02.04.2020.A366). Pregnant NMRI mice were purchased from Charles River Laboratories (Sulzfeld, Germany, RRID:IMSR_CRL:605). CD‐1 mice (RRID:IMSR_CRL:022) were used for functional experiments using SEC protein fractions and RNA depletion, following initial validation of observed phenotypes. Dissections were performed as described in ref [6]. Briefly, dissociated cortical and hippocampal neurons were seeded onto cell culture dishes or nitric acid‐washed glass coverslips coated with 0.5 mg/mL poly‐D‐lysine (Sigma) in Basal medium Eagle's containing Eagle's salts, 0.45% glucose, 10% horse serum (Capricorn; HOS‐1A), 1 mm sodium pyruvate, 100U/mL Penicillin, and 0.1 mg/mL Streptomycin. Plating medium was replaced with serum‐free Neurobasal Plus medium supplemented with 2% B27 Plus supplement (Gibco), 2 mm Glutamax, 100U/mL Penicillin and 0.1 mg/mL Streptomycin after 3 h. For subsequent experiments, AAV transduction was performed at 0DIV during replacement of plating medium and AAVs were washed off at 3DIV. AAVs were added at an MOI of 10 000. Cells were supplemented with fresh complete Neurobasal Plus medium every 3–5 days. For EV treatment of hippocampal neurons, EVs from 7DIV cortical neurons isolated by SEC were added to sister cultures of primary hippocampal neurons for 3 days, at a ratio of 12.5:1 donor to recipient cell. Cell transfection was performed using lipofectamine 2000 (Thermo Fisher) for plasmid DNA or lipofectamine RNAiMAX (Thermo Fisher) for siRNAs, according to manufacturer's instructions. Neurons were transfected on 5–6DIV. For KD experiments, neurons transfected with siRNAs were harvested 72 h after transfection.

### 4.3 EV Isolation

For all experiments, 16 h before EV isolation conditioned medium was aspirated and cells were washed with plain neurobasal medium or 1x PBS for cortical neurons (7DIV) or N2a cells, respectively. EV collection medium was neurobasal medium (with Pen‐strep, Glutamax and B27) for neurons or DMEM (Gibco, with Glutamax and Pen‐strep, without FBS) for N2a cells. All centrifugation steps were performed at 4°C, unless otherwise stated. The cell culture supernatant was subjected to serial centrifugation at 3500 x*g* for 10 min, followed by two times 4500 x*g* for 10 min, and 10 000 x*g* for 30 min.

#### 4.3.1 Size Exclusion Chromatography (SEC)

For RNA isolation from EVs or EV treatment on neurons, samples after serial centrifugation were added to the SEC column (qEVoriginal 70 nm, IZON Bioscience) and processed as per manufacturer's instructions. Fractions 7–12 were collected and pooled. When small sample volume was required, the sample was further concentrated by ultracentrifugation at 100 000 x*g* for 1h using the TLA100.3 rotor in the Optima MAX‐XP tabletop ultracentrifuge (Beckman Coulter).

#### 4.3.2 Ultracentrifugation

For Western Blot, EVs were isolated by ultracentrifugation. Following serial centrifugation, 10 000 x*g* supernatants were subjected to ultracentrifugation at 100 000 x*g* for 1 h (as above). Following the first ultracentrifugation step, EV pellets were washed in cold 1x PBS and subjected to a second ultracentrifugation step.

#### 4.3.3 Density Gradient Centrifugation

EVs derived from 16 million cortical neurons (6 DIV) were first isolated by ultracentrifugation at 100 000 x*g* as described above and resuspended in HEPES‐buffered saline including EDTA‐free protease inhibitor cocktail (Roche). For the gradient, 30% (5 mL), 20% (2.2 mL), and 10% (2 mL) iodixanol solutions were freshly prepared using a 50% working solution of iodixanol containing 0.25 m sucrose, 1 mm EDTA, and 10 mm HEPES pH7.5 and protease inhibitors (Roche). Re‐suspended EVs were layered underneath the gradient. 0.2 mL of HEPES buffer was layered on top of the gradient prior to centrifugation at 120 000 x*g* for 18 h with maximum acceleration and deceleration in a swinging bucket rotor (SW41 Ti). Ten equal volume fractions were collected from top to bottom, diluted in 3 mL 1x PBS, and centrifuged again at 100 000 x*g* for 1 h (TLA100.3). The resulting EV pellets were processed for western blotting or RNA extraction.

#### 4.3.4 Immunoprecipitation

Following serial centrifugation (as described above), the resulting 10 000 x*g* supernatant was incubated overnight with CD81/63/9 antibody‐coupled beads (EV isolation kit pan mouse, Miltenyi Biotec) with rotation at 4°C, and processed according to manufacturer's protocols.

#### 4.3.5 RNA Depletion of EVs

For depletion of EV‐RNA cargo, 10 000 x*g* supernatants were treated with a final concentration of 0.2% Saponin and 10 µg/ml RNase A enzyme and incubated in a 37°C incubator for 20 min prior to purification using SEC and subsequent ultracentrifugation.

### 4.4 Nanoparticle Tracking Analysis

NTA was performed on NanoSight NS500 (Malvern Panalytical, Malvern, UK) equipped with a 488 nm. Before measurement, only PBS buffer (0.2 µm filtered) was loaded, recording parameters were set and buffer checked for absence of any particles. EV samples were then diluted in PBS at a 1:1 ratio to reach the optimal range of particles in the field of view. Five 60‐s measurements per sample were recorded at 25°C and then analyzed with same parameters using NTA analysis software version NTA 3.1 Build 3.1.54. Videos containing very large particle aggregates leading to big chunks or flares, if observed, were excluded as they affected the tracking‐analysis of the main nanoparticle population.

### 4.5 Protein Analysis

Cells were washed three times with cold PBS, lysed with CHAPS buffer (1% CHAPS, 5 mm EDTA, 50 mm Tris‐HCl, pH8) containing EDTA‐free protease inhibitor cocktail (Roche) and kept on ice for 15 min. Lysate was centrifuged at 13 000 x*g* for 15 min at 4°C and the supernatant was collected.

#### 4.5.1 Immunoprecipitation

For protein‐protein interaction assays, cells were washed three times with cold PBS on ice and lysed in buffer containing 20 mm Tris‐HCL pH7.5, 150 mm NaCl, 2.5 mm MgCl_2_, 0.5% NP‐40, and EDTA‐free protease inhibitor cocktail (Roche). For RNA‐protein interaction assays, the lysis buffer included 10 mm HEPES pH7.3, 50 mm KCl, 5 mm MgCl_2_, 0.5% NP‐40, 0.5 mm DTT, EDTA ‐free protease inhibitor cocktail (Roche), and 1U/µL SuperaseIn (Invitrogen), prepared in DEPC‐treated water. Cell lysates were kept on ice for 15 min followed by centrifugation at 13 000 x*g* for 15 min at 4°C. Pull‐down antibodies were conjugated with magnetic Dynabeads Protein A for rabbit antibodies (anti‐GFP; Abcam # ab290, RRID:AB_303395, anti‐TRBP; Proteintech # 15753_1_AP, RRID:AB_2200485) or Dynabeads Protein G for mouse antibodies (anti‐myc; Sigma Aldrich # M4439, RRID:AB_439694, anti‐HA; Sigma Aldrich # H9658, RRID:AB_260092) for 30 min with rotation and according to manufacturer's instructions prior to incubation with cell lysate supernatants. As control, normal rabbit IgG (Cell Signaling # 2729, RRID:AB_1031062) or mouse anti‐IgG (Biomol # D609‐0200, RRID:AB_3752996) antibodies were used. The mixture was then incubated on a rotating wheel for 16 h at 4°C. Beads were washed four times with the corresponding IP buffer and incubated in Laemmli buffer or QIAzol lysis reagent for Western Blot or RNA extraction respectively.

#### 4.5.2 Immunoblotting

Cell lysates or EV pellets were mixed with Laemmli sample buffer by vortexing and heated at 70°C for 10 min before loading onto SDS‐PAGE gel for electrophoresis. After that, proteins were transferred onto polyvinylidene difluoride (PVDF) membranes. The membranes were blocked in 5% semi‐skimmed milk powder in Tris‐buffered saline containing 0.1% Tween‐20. Primary and secondary antibodies were diluted in blocking buffer. The following primary antibodies were used: anti‐GFP (Invitrogen # A6455, RRID:AB_221570), Ago2 (Cell Signaling # 2897T, RRID:AB_2096291), Alix (BioLegend # 634501 and # 634502, RRID:AB_2268110, RRID:AB_2162471), Calnexin (Sigma Aldrich # C4731, RRID:AB_476845), Dicer (Sigma Aldrich # SAB4200087, RRID:AB_10603336), Flotilin 2 (Santa Cruz # sc‐25507, RRID:AB_2278617), HA (Sigma Aldrich # H9658, RRID:AB_260092), hnRNPA2B1 (Abcam # ab6102, RRID:AB_305293), Myc (Cell Signaling # 2276, RRID:AB_331783), PACT (Abcam # ab75749, RRID:AB_1310539), Syncrip (Abcam # ab184946, RRID:AB_3675486), TRBP (Abcam # ab180947, RRID:AB_3675488), YBX‐1 (Origene # TA806164S, RRID:AB_2627995). HRP‐conjugated secondary antibodies were purchased from JacksonImmuno Research (anti‐mouse IgG; # 115‐035‐062, RRID:AB_2338504, anti‐rabbit IgG; # 111‐035‐003, RRID:AB_2313567). Blots were detected using the ChemiDoc MP imaging system (Bio‐rad).

### 4.6 Proteomics

Mass spectrometry and proteomics analysis of neuronal EVs was previously published (ref [22]). For mass spectrometry of TRBP immunoprecipitates, primary neurons from rat embryonic primary neurons (as in ref [20]). were lysed at 7DIV using lysis buffer containing 0.5% NP‐40, 300 mm NaCl, 5 mm MgCl_2_, and 50 mm Tris pH7.5 and immunoprecipitated using 2 µg of TRBP (Abcam # ab129325, RRID:AB_11156300) or normal mouse IgG control antibody (Santa Cruz Biotechnology # sc‐2762, RRID:AB_737179) coupled to Dynabeads Protein G, as described above. The beads were separated on a magnet and washed four times with 100 mM ammonium bicarbonate to remove detergent and protease inhibitors.

For elution, 100 µL elution buffer 1 (100 mm ammonium bicarbonate, 1 m urea, 1 µg trypsin (Promega) was added to each sample. After 30 min incubation at 30°C, the supernatant containing the digested proteins was collected. Beads were washed twice with elution buffer 2 (100 mm ammonium bicarbonate, 1 m urea, 5 mm Tris(2‐carboxyethyl) phosphine (TCEP), Sigma–Aldrich) and added to the first elution fraction. Digestion was allowed to proceed overnight at 30°C. Following digestion, the peptides were incubated with 10 mm iodoacetamide (Sigma–Aldrich) for 30 min at 25°C in the dark.

Peptides were acidified and desalted on C18 solid phase extraction cartridges (Macherey‐Nagel). The cartridges were prepared by adding acetonitrile (CAN), followed by equilibration with 0.1% TFA (trifluoroacetic acid). Peptides were loaded on equilibrated cartridges, washed with 0.1% TFA and finally eluted with 50% ACN/ 0.1% TFA, dried and reconstituted in 0.1% TFA. The following liquid chromatography‐mass spectrometry (LC‐MS) analysis was carried out on a Q‐Exactive Plus instrument connected to an Ultimate 3000 RSLC nano and a nanospray flex ion source (all Thermo Scientific). Peptide separation was performed on a reverse‐phase HPLC column (75µm × 42 cm) packed in‐house with C18 resin (2.4 µm, Dr. Maisch). The following separating gradient was used: 98% solvent A (0.15% formic acid) and 2% solvent B (99.85 acetonitrile, 0.15% formic acid) to 10% solvent B over 6 min, to 32% for 95 min, and to 50% for additional 35 min at a flow rate of 300nL/min. The data acquisition mode was set to obtain one high‐resolution MS scan at a resolution of 70,000 full width at half maximum (at m/z 200) followed by MS/MS scans of the 10 most intense ions. To increase the efficiency of MS/MS attempts, the charged state screening modus was enabled to exclude unassigned and singly charged ions. The dynamic exclusion duration was set to 30s. The ion accumulation time was set to 75 ms for MS and 50 ms at 17 500 resolution for MS/MS. The automatic gain control was set to 3 × 106 for MS survey scans and 1 × 105 for MS/MS scans.

For spectral based assessment searches were carried out using Mascot (v2.5, Matrix Science) [50] with 10 ppm MS1 and 0.02 Da fragment ion tolerance with Carbamidomethylation (C) as fixed, oxidation (M) and phosphorylation (S, T, Y) as variable modifications. Search results were evaluated in Scaffold 4 (Proteome Software) [51].

### 4.7 Immunofluorescence

Cells were fixed in 4% PFA/ 4% sucrose / 1xPBS solution for 15–20 min at room temperature and washed three times with 1x PBS. Next, cells were permeabilized with 0.1% Triton X‐100 in PBS for 5 min at room temperature. Primary and secondary antibodies were diluted in 2% BSA / 1xPBS/ 0.1% Tween‐20. Primary antibodies (anti‐Homer1; Synaptic Systems # 160 003, RRID:AB_887730), Rab7; Abcam # ab137029, RRID:AB_2629474), Synapsin1/2; Synaptic Systems # 106 002, RRID:AB_887804), Syncrip; Abcam # ab184946, RRID:AB_3675486) were incubated overnight at 4°C in a humidified chamber. Secondary antibodies (Alexa‐Fluor 488; Thermo Fisher Scientific # A78948, RRID:AB_2921070, Alexa‐Fluor 647; Thermo Fisher Scientific # A‐31573, RRID:AB_2536183, Cy3; Abcam # ab97145, RRID:AB_10679516) were used at a dilution of 1:2000 and incubated for 45min‐1 h at room temperature. For immunostainings of intracellular membrane organelles, 0.25% saponin instead of Triton‐X100 was used for permeabilization. Here, coverslips were incubated in 5% BSA/ 0.25% saponin/ 1xPBS for 30 min at room temperature followed by incubation of primary and secondary antibodies diluted in the same solution. 0.25% Saponin was also added to 1xPBS used in the washing steps after antibody incubation. Coverslips were mounted on slides using 10% Mowiol 4–88 containing 2.4% DABCO (*1,4‐Diazabicyclo[2.2.2]‐octane*).

For proximity ligation assay, cells were fixed and permeabilized using 0.1% Triton X‐100 as above. Subsequent steps were carried out using kit reagents (Duolink In situ detection reagents red; # DUO92008, anti‐rabbit PLUS; # DUO92002, RRID:AB_2810940, anti‐mouse MINUS; # DUO92004, RRID:AB_2713942) and according to manufacturer instructions.

### 4.8 Image Analysis

#### 4.8.1 Co‐Localization Analysis

XY images were acquired under 63x /1.40 NA oil‐immersion objective on an LSM800 Zeiss microscope. For analysis of GFP‐Syncrip and mCherry‐TRBP/ mCherry‐Climp63 co‐localization in N2a cells the Fiji [52] JaCoP co‐localization plugin was used, using a cell mask as the region of interest (ROI), which was generated based on the MaxEntropy algorithm in Auto‐threshold function. Fiji thresholding and the JaCoP plugin was also used for co‐localization analysis of GFP‐Ago2‐Syncrip‐ORP1L and Protrudin‐Syncrip‐Rab7 images. For analysis of Syncrip co‐localization in neuronal dendrites from ORP1L or Protrudin‐expressing neurons, XYZ Airyscan images were acquired on Zeiss LSM880 and co‐localization analysis was performed using the Coloc extension of Imaris 9.7 software. Optimal channel thresholding was performed for each channel and kept constant across conditions and experimental trials. Channel segmentation was performed using *Surfaces* for visualization purposes.

For analysis of RNA granule overlap, XYZ images were acquired using the 63x oil‐immersion objective of the Zeiss LSM900 confocal microscope and processed by deconvolution on Zen 3.11 software. Maximum intensity projection images were then inverted to grayscale and the number of puncta per cell was counted manually on Fiji. Only cells with similar expression levels were selected for analysis, prior to counting.

#### 4.8.2 Quantification of Synapse Density

XYZ images of dendritic regions were captured using the 63x objective of LSM880 for EV‐donor neurons and the LSM800 for EV‐recipient neurons and analyzed in Imaris 10.2. First, a co‐localization channel between Homer and Synapsin was created using the Coloc extension. Following segmentation using the *Surfaces* module, the total volume of co‐localized puncta or Synapsin within 2 µm of GFP‐ or dsRed‐labeled dendrites was quantified. Data were normalized to dendrite length or dendrite volume for donor neurons and recipient neurons, respectively. For experiments using RNA‐depleted EVs and non‐EV fractions, xyz images were acquired using a Leica DMi8 confocal microscope. Co‐localization analysis was performed using the Coloc2 plugin in Fiji software in 50µm‐long dendritic regions using a region of interest (50 × 21.85 µm) and the GFP channel as a mask.

#### 4.8.3 Quantification of Dendrite Complexity

For dendrite complexity experiments, XYZ images of dsRed‐expressing neurons were acquired using a 20x/0.8 M27 objective on LSM880. Neuronal tracing of dendrites was performed using the SNT plugin of the Fiji Neuroanatomy tool box. For Sholl analysis, concentric circles were placed from the soma of the neuron at an interval of 15 µm and the number of intersections were quantified.

### 4.9 RNA Isolation and rt‐qPCR

Total RNA was isolated from EVs or cell lysates using QIAzol (QIAGEN). The concentration of RNAs was determined by Nanodrop spectrophotometer (DeNovix). Samples were either stored immediately in −80°C or reverse transcribed to cDNA.

For miRNA quantification, total RNA was purified using the miRNeasy Micro Kit (QIAGEN, # 217084) with an extra step of DNase I treatment by RNase Free DNase Set (preAnalytiX, # 79254). Isolated miRNAs were reverse transcribed into cDNAs by TaqMan Advanced miRNA cDNA Synthesis Kit (Thermo Fisher, # A28007). qPCR was then carried out on StepOnePlus Real‐Time PCR system (Applied Biosystems) using the TaqMan Fast Advanced Master Mix (Thermo Fisher, # 4444557) and TaqMan assays (# A25576, mmu‐let‐7c‐5p; Assay ID: 478577_mir, mmu‐miR‐27b‐3p; Assay ID: mmu478270_mir, mmu‐miR‐204‐5p; Assay ID: 478491_mir) according to manufacturer instructions.

For TRBP KD confirmation, total RNAs from neurons were extracted. cDNAs were obtained by reverse transcription of RNAs with SuperScript IV VILO Master Mix kit with ezDNase (Invitrogen, # 11766050). RT‐qPCR was performed with TRBP mRNA primers and SYBR green PCR kit (Thermo Fisher, # 4367659) on StepOnePlus Real‐Time PCR system.

### 4.10 Small RNA Sequencing

Sixteen small RNA libraries representing four biological replicates from control or TRBP KD neuronal cell lysates and corresponding EVs were prepared at Novogene using the Plant and Animal Exosome small RNA‐seq (WOBI) protocol and sequenced using Illumina SE50 (20 M raw reads per sample). Raw data was processed by nf‐core/smrnaseq analysis pipeline [53] for quality control and alignment based on mature miRNA sequences from miRbase (https://www.mirbase.org/) [54]. Differentially expressed miRNAs were identified based on statistical analysis by EdgeR [55] and limma [56] packages. In further analysis, differentially expressed miRNAs are plotted on volcano plots and heatmap according to the significance and fold changes with R packages including ggplot2 [57] ComplexHeatmap [58].

For motif enrichment analysis, enriched motifs were identified using HOMER (Hypergeometric Optimization of Motif EnRichment) [59]. To examine enriched motifs in EV samples, CL miRNA was used as background. Motifs in TRBP‐regulated miRNAs (*p*<0.05) were identified using EV or CL as background respectively. MiRNAs were annotated according to edgeR (mean_cpm > 1) analysis, and turned into 22 nt by either trimming or insertion of U at the 3’ end, to enable enrichment analysis.

### 4.11 Gene Ontology and Pathway Analysis

Gene ontology and KEGG pathway enrichment analysis for differentially expressed miRNAs were performed based on miRDB [54] target prediction analysis (target score ≥80) for mouse miRNAs using the mouse primary neuron transcriptome as background (based on ref [6]). The analyses were performed by clusterProfiler package [60], including ego_simple function to reduce the redundancy. Synaptic gene ontology was performed in SynGO using differentially expressed miRNAs that also contain Syncrip binding motifs (GGC[A/U]). miRDB database was also used for gene prediction with the same target threshold. For proteomics datasets, genes in TRBP immunoprecipitates were converted to their mouse homologues prior to functional enrichment analysis. Only genes with at least 2 peptide counts and three counts more than IgG control were used for analysis. Functional enrichment analysis of genes common to TRBP immunoprecipitates and EVs was performed using *STRING* version 12 (www.string‐db.org).

### 4.12 Quantification and Statistical Analysis

Data are represented as mean± standard deviation, unless otherwise stated. Statistical significance tests were performed using GraphPad Prism software (v10.6.1) as indicated in corresponding figure legends. All imaging experiments were performed in a blinded manner. At least 10 cells per experimental condition and in at least 3 biological trials were used for analysis. R statistical language (v4.5.1) and R packages (EdgeR and limma) were used for analysis of RNA sequencing data.

## Author Contributions

Conceptualization, Y.Z., A.A.; methodology, Y.Z., S.L., A.S, M.O., H.S., T.G., A.A.; formal analysis, Y.Z., S.L., A.S., T.G.; data curation, Y.Z., T.G.; writing, Y.Z., A.A.; funding acquisition, M.O., A.A.

## Ethics Statement

Preparation of mouse primary neurons was performed with adherence to local Animal Welfare regulations (LANUK NRW, approval number: 81‐02.04.2020.A366).

## Conflicts of Interest

The authors declare no conflicts of interest.

## Supporting information




**Supporting File 1**: advs78067‐sup‐0001‐SuppMat.docx.

**Supporting File 2**: advs78067‐sup‐0002‐Table1.xlsx.

**Supporting File 3**: advs78067‐sup‐0003‐Table2.xlsx.

**Supporting File 4**: advs78067‐sup‐0004‐Table3.xlsx.

**Supporting File 5**: advs78067‐sup‐0005‐Table4.xlsx.

**Supporting File 6**: advs78067‐sup‐0006‐Table5.xlsx.

## Data Availability

Small RNA sequencing data generated in this study have been deposited in Gene Expression Omnibus (GEO, GSE319765). Mass spectrometry proteomics data have been deposited to the ProteomeXchange Consortium via the PRIDE (https://pubmed.ncbi.nlm.nih.gov/39494541/) partner repository with the dataset identifier PXD075087. All source data are available upon request.
