# Supplementary material for: The Cytoplasmic MicroRNA‐Production Complex Regulates MicroRNA Sorting to Extracellular Vesicles During Synaptogenesis
Source: Adv Sci (Weinh). 2026 Sep 30:e78067. Online ahead of print. doi: 10.1002/advs.78067 (PMC13626668; doi:10.1002/advs.78067)
Supplement: Supplementary file 1 [file ADVS-9999-e78067-s005.docx]

**Zeng et al ‘*The cytoplasmic microRNA-production complex regulates microRNA sorting to extracellular vesicles during synaptogenesis’***

**Supplementary Figures**

**Figure S1**

**Figure S1: The Dicer co-factor TRBP interacts with RBPs secreted in EVs. A)** Gene ontology analysis for the biological processes of all overlapping proteins in TRBP IP and EV mass spectrometry datasets. Analysis was performed in *String*. **B)** GFP-PACT and GFP-TRBP bind to hnRNPA2B1. N2a cells were co-transfected with myc-hnRNPA2B1 and GFP-PACT or GFP-TRBP and IP was performed using an anti-myc antibody. **C)** GFP-TRBP binds to myc-hnRNPA2B1 in an RNA-dependent manner. GFP IP were treated with RNAse A (0.1 µg/ml, 10 min, 37 °C) or control. **D)** TRBP overexpression does not change the abundance of Syncrip or hnRNPA2B1 in N2a cells and EVs. N2a cells were transfected with GFP, GFP-TRBP-WT or -∆C and medium and small EVs were isolated using UC at 10,000 x*g* and 100,000 x*g*, respectively.

**Figure S2**

**Figure S2: TRBP bi-directionally regulates intracellular and EV-miRNA abundance. A)** Western blot of neuronal cell lysates (CL) transfected with siCtrl or siTRBP as depicted. **B)** Relative abundance of TRBP immunoblots as in **A**, n=3, ***p*=0.009, 1-sample t-test. **C)** Relative abundance of TRBP mRNA in neurons transfected wth siCtrl or siTRBP quantified by rt-qPCR, n=5; ***p*=0.004; 1-sample t-test. The same samples and corresponding EVs were sent for small RNA sequencing. **D)** Heatmap demonstrating the abundance of differentially expressed miRNAs in CL. **E)** Gene ontology of the predicted targets of differentially expressed miRNAs in CL (p<0.05 and |logFC>0.3|).

**Figure S3**

**Figure S3: A TRBP binding-deficient mutant of Syncrip shows decreased binding to TRBP-regulated miRNAs with a Syncrip-like motif. A)** Motif enrichment analysis for miRNAs in EV versus CL samples. **B)** PACT interacts with Syncrip-WT, -∆N and -∆C. N2a cells were transfected as indicated and IP was performed with anti-GFP antibody. **C)** PACT binds more strongly to the Syncrip-∆C mutant. Analysis of PACT relative intensity in IP versus input lysates as in **B,** n=4, **p*=0.02, Kruskal Wallis test. **D)** Confocal images of N2a cells expressing GFP-Syncrip-WT, -∆N or -∆C and mCherry-TRBP, scale bars; 5µm. **E)** Mander’s co-localization coefficients in the cytoplasm of images as in **D**, n= 66-76 cells, *****p*<0.0001, 1way ANOVA. **F-H)** Intracellular abundance of candidate miRNAs, n=3*.* **I)** Total RNA concentration of small EVs isolated by SEC in N2a cells expressing GFP Ctrl, GFP-Syncrip WT, ∆N or ∆C, n=3, 1way ANOVA.

**Figure S4**

**Figure S4: The C-terminal TRBP-binding domain of Syncrip mediates its localization to RNA granules. A-B)** Quantification of relative abundance of GFP (**A)** and Dicer (**B**) in HA-IP in cells expressing HA-Ago2 and GFP, GFP-Syncrip WT, ∆C or ∆N, n=3, *p=0.04, ****p<0.0001, 1way ANOVA. **C)** GFP-Syncrip-WT and -∆N, but not -∆C, form puncta that resemble RNA granules. Inverted microscopy images of N2a cells expressing, scale bar; 15 µm. **D)** Syncrip is present in P bodies. N2a cells were transfected with mCherry-Syncrip-WT, -∆N or -∆C and GFP-Dcp1a. Arrows point to overlapping puncta, scale bar; 5 µm.

**Figure S5**

**Figure S5: ER-endosome MCS regulate the interaction between Syncrip and TRBP. A)** Overlap of GFP-Syncrip-WT, -ΔN and -ΔC with ER marker mCherry-Climp63 in N2a cells, scale bar; 5 µm. **B)** Mander’s co-localization co-efficients of images as in **A**, n=70-83 cells, *****p*<0.0001, 1way ANOVA. **C)** Airyscan images of 7DIV primary hippocampal neuron dendrites expressing GFP-Protrudin-WT or -FYVE4A and mCherry-Syncrip and immunostained with Rab7 antibodies. Arrows depict puncta overlap, scale bar; 15 µm.

**Figure S6**

**Figure S6: Endosome MCS regulate the localization of Syncrip and Ago2 to endosomes. A)** Airyscan images of primary hippocampal neurons (7DIV) expressing mCherry-ORP1L-WT, -∆ORD or -∆ORDPHDPHD and GFP-PACT, and immunostained with Syncrip. **B)** Airyscan images of primary hippocampal neurons (7DIV) expressing mCherry-ORP1L-WT, -∆ORD or -∆ORDPHDPHD and GFP-Ago2, and immunostained with Syncrip antibodies, scale bar; 5µm. **C)** Schematic depicting proposed model. Created in BioRender. Antoniou, A. (2026) https://BioRender.com/am6gafk

**Figure S7**

**Figure S7: Syncrip overexpression does not significantly change dendrite outgrowth or EV secretion. A)** Inverted images of EV recipient neurons expressing dsRed and treated with EV Ctrl, or EVs from GFP-Syncrip-WT, -∆N and -∆C expressing neurons, scale bar; 20 µm. **B)** Nanoparticle tracking analysis of SEC-isolated EVs from neurons transduced with GFP-Syncrip or Ctrl, as in **A**, n=9-13 measurements from 3 independent experiments, dashed line represents standard error. **C-D)** Median size and particle concentration of nanoparticle tracking analysis as in A, n=3.

**Figure S8**

**Figure S8: Differential regulation of synapse formation in donor and recipient neurons by Syncrip WT and mutant overexpession. A-B)** Representative Airyscan images and reconstructed 3-dimensional surfaces of synaptic puncta and dendrites by Imaris for donor (**A**) and recipient (**B**) cells. The surface of Synapsin or Homer1/Synapsin co-localization puncta is filtered to be within 2µm from dendrites. Scale bar 2µm. **C**) Total RNA concentration in EVs following Saponin-RNase A treatment or control vehicle. **D-E**) Representative images of recipient neurons treated with RNA-depleted EVs (**D**) or non-EV protein fractions isolated by SEC-UC (**E**).

**Supplementary Methods**

**List of primers and oligonucleotides**

| **Name** | **Company** | **Sequence or Catalogue number** |
| --- | --- | --- |
| GFP-SYNCRIP-ΔC | Sigma-Aldrich | Fw; ccggtcctgacctgttacaatgag,  Rev; gagcttttctttctttcctcttctg |
| GFP-SYNCRIP-ΔN | Sigma-Aldrich | Fw; ccttctgttggcactgagatatttg,  Rev; gtaagtcttcattgctcgagatctg |
| pAAV-GFP-SYNCRIP-WT | Sigma-Aldrich | Fw; taagatccgctagcgctaccggtcg,  Rev; tataagcttcgtcgactgcagaattctca |
| pAAV-GFP-SYNCRIP-ΔC | Sigma-Aldrich | Fw; taagatccgctagcgctaccggtcg,  Rev; tataagcttcgtcgactgcagaattctca |
| pAAV-GFP-SYNCRIP-ΔN | Sigma-Aldrich | Fw; taagatccgctagcgctaccggtcg,  Rev; tataagcttcgtcgactgcagaattctca |
| Myc-TRBP | Sigma-Aldrich | Fw; agcttatggaacagaaa, ctgatctctgaagaagacctgg,  Rev; agagatcagtttctgttccata, gatcccaggtcttcttc |
| Myc-hnRNPA2B1 | Sigma-Aldrich | Fw; tatggatccatgtctaagtcagagtc,  Rev; atactcgagttaaaatcttctgccac |
| siRNA Ctrl | Sigma-Aldrich | MISSION siRNA Universal Negative Control, Cat#SIC003 |
| TRBP siRNA | Horizon Discovery | ON-TARGETPlus SMARTPool Cat#L-049304-01-0005 |
